# Thermosonication of traditional poppy vinegar: modulation of biomolecules, phenolic profile, and antidiabetic potential in a functional fermented food

**DOI:** 10.3389/fnut.2026.1828372

**Published:** 2026-04-30

**Authors:** Meryem Akhan, Melikenur Türkol, Enes Açıkgözoğlu, Şeyma Varınca Yıldız, Mehmet Ali Şimşek, Remzi Gürfidan, Nazan Tokatlı Demirok, Seydi Yıkmış, Emad Karrar, Moneera O. Aljobair, Isam A. Mohamed Ahmed

**Affiliations:** 1 Department of Nutrition and Dietetics, Istanbul Esenyurt University, Istanbul, Türkiye; 2 Nutrition and Dietetics, Faculty of Health Sciences, Tekirdag Namik Kemal University, Tekirdag, Türkiye; 3 Department of Database, Network Design and Management, Isparta Vocational School of Information Technologies, Isparta University of Applied Sciences, Isparta, Türkiye; 4 Department of Tumor Biology and Immunology, Institute of Health Sciences, Tekirdag Namik Kemal University, Tekirdag, Türkiye; 5 Department of Software Engineering, Faculty of Engineering and Natural Sciences, Bandırma Onyedi Eylül University, Balikesir, Türkiye; 6 Department of Food Technology, Tekirdag Namik Kemal University, Tekirdag, Türkiye; 7 Department of Plant Sciences, North Dakota State University, Fargo, ND, United States; 8 Department of Sports Health, College of Sports Sciences and Physical Activity, Princess Nourah Bint Abdulrahman University, Riyadh, Saudi Arabia; 9 Department of Food Sciences and Nutrition, College of Food and Agricultural Sciences, King Saud University, Riyadh, Saudi Arabia

**Keywords:** antidiabetic potential, biomolecules, functional fermented food, molecular docking, phenolic profile, thermosonication, traditional poppy vinegar

## Abstract

Traditional poppy vinegar is a functional fermented food matrix enriched with diverse biomolecules that may contribute to metabolic health. In this study, the effects of thermosonication on the biomolecular profile, antioxidant characteristics, and antidiabetic potential of traditionally produced poppy (*Papaver rhoeas* L.) vinegar were investigated using an integrated experimental and modeling approach. Thermosonication was performed at 26 kHz and 200 W, while treatment time (8–16 min), amplitude (60–100%), and temperature (40–60 °C) were optimized through a Box–Behnken design (*n* = 15). The primary responses were inhibition of α-glucosidase and α-amylase. Among the tested models, the Ridge (Poly2) model showed the best predictive performance, with *R*^2^ values of 0.9414 for α-glucosidase inhibition and 0.7951 for α-amylase inhibition, together with low LOOCV-based MAPE values. Multi-objective optimization identified 13.86 min, 84.68% amplitude, and 47.40 °C as the optimum conditions, yielding predicted inhibition values of 42.34% for α-glucosidase and 37.96% for α-amylase; the experimental validation results were 41.73 and 36.11%, respectively. Thermosonication better preserved the functional quality of poppy vinegar than conventional pasteurization. Specifically, the thermosonicated samples exhibited a remarkably high retention of free radical-scavenging activity (DPPH) alongside greater levels of total phenolics, flavonoids, and anthocyanins. The preservation of this robust antioxidant capacity is of great significance, as it prevents the thermal degradation typically induced by conventional processing, thereby sustaining the structural integrity of bioactive compounds and maximizing the overall health-promoting potential of the vinegar. Phenolic profiling further demonstrated treatment-dependent changes in individual compounds, with quercetin detected in the thermosonicated sample. Molecular docking analysis supported the mechanistic plausibility of enzyme inhibition by showing favorable binding affinities of selected phenolics, particularly quercetin, toward α-amylase. Overall, the findings indicate that thermosonication is a promising green processing strategy for modulating biomolecules and preserving the functional potential of traditional poppy vinegar as a fermented food with antidiabetic relevance.

## 1 Introduction

Edible flowers have been incorporated into human diets since ancient times. Recently, consumer interest has surged due to their rich nutritional profiles and diverse bioactive constituents that offer potential health-promoting benefits (1, 2). Furthermore, they are highly valued in gastronomy for imparting unique aromas, exquisite flavors, and vibrant colors to culinary dishes (3). It is predominantly grown in temperate zones across northern Europe, Africa, the Americas and western Asia (4). The Papaver plant, which is scientifically named *Papaver rhoeas L*., belongs to the family *Papaveraceae* (5). There are 16 species of the genus Papaver, which is also known in Turkish as “gelincik,” in the European Flora (6). Bioactive components are present in this important edible plant, which is called poppy (7).

Vinegar is a fermented product characterized by significant technological relevance and nutritional importance (8). Worldwide, vinegar varieties are broadly divided into two main groups based on the type of substrate used in their production: fruit-based vinegars and grain-based vinegars (9). Fruit-based vinegars (such as apple, grape, and date vinegar) are produced by subjecting raw materials with high sugar content to alcoholic fermentation followed by acetic acid fermentation (10, 11). Vinegar is attracting increasing industrial interest. This is due to its nutritional, sensory and bioactive properties (8). A comprehensive analysis of various fruit vinegars revealed their diverse phenolic and organic acid profiles, including gallic, chlorogenic and caffeic acids, and demonstrated their significant antioxidant capacities (12, 13).

The thermosonication process, achieved by combining ultrasound treatment in a water bath with gentle heating, is a particularly promising method. In this process, localized temperature increases and intense mechanical shear forces are induced by acoustic cavitation (14, 15). This process relies on acoustic cavitation, the formation and collapse of microbubbles in a liquid medium, which results in localised temperature increases and intense mechanical shear forces (16). These effects disrupt cellular structures, promote the release of bioactive substances, and homogenize (17, 18).

Compared to conventional thermal processing methods used in common today, such as pasteurization, thermosonication offers distinct advantages. Conventional thermal treatments often result in the irreversible degradation of heat-sensitive (thermo-labile) biomolecules and a reduction in functional properties. In contrast, the mechanical forces generated by ultrasonic cavitation in thermosonication disrupt the cellular matrix, facilitating the release of intracellular constituents and enhancing the extraction efficiency of secondary metabolites without the need for extreme heat. This processing strategy is advantageous for preserving the phenolic profile, antioxidant capacity, and antidiabetic potential of functional foods compared to traditional pasteurization methods (14, 19, 20).

Understanding the multifaceted, nonlinear interactions among processing time, application temperature, and amplitude level is crucial. Machine learning methods, however, can successfully model these complex relationships in large and multidimensional datasets and generate reliable predictions. Machine learning models such as Ridge Regression, Support Vector Regression (SVR), and Bayesian Ridge Regression have been successfully used in food science for modeling various processes, estimating quality parameters, and conducting optimization studies (21–23). The biological activities of phenolic compounds found in vinegar are fundamentally governed by their molecular-level interactions with target enzymes. In this context, molecular docking is a critical computational approach that enables the prediction of binding modes and noncovalent interaction affinities within protein targets at atomic resolution. By integrating these in silico analyses, our study aims to elucidate the potential molecular interactions between the predominant phenolic compounds and alpha-amylase.

To the best of our knowledge, no study has simultaneously investigated the effects of thermosonication on the phenolic profile, antioxidant capacity, and antidiabetic activity of poppy vinegar using machine learning and molecular docking. The effects of thermosonication parameters on α-glucosidase and α-amylase were evaluated using machine learning models (Ridge, support vector, and Bayesian regression), and control, thermosonicated, and pasteurized poppy vinegar samples were comparatively examined. The total phenolic content (TPC), total flavonoid content (TFC), antioxidant capacities (DPPH and TAC), and profiles of individual phenolic components were determined to assess treatment-induced changes in bioactive composition. α-glucosidase is a key intestinal enzyme responsible for the final step of carbohydrate digestion by hydrolyzing α-1,4-glycosidic linkages to release glucose, thereby contributing directly to postprandial blood glucose elevation; inhibition of this enzyme delays glucose release and absorption, representing an effective strategy for glycemic control. To elucidate the observed biological effects at the molecular level, interactions between the main phenolic compounds and diabetes-related target enzymes were evaluated using in silico molecular docking; in this context, α-amylase was selected as a representative carbohydrate-hydrolyzing enzyme due to the availability of a well-characterized crystal structure and its widespread use in food bioactivity studies, whereas docking analysis for α-glucosidase was not included due to the limited availability of high-resolution and structurally consistent models, which may affect the reliability of in silico predictions, and therefore the docking results are presented as a supportive mechanistic model rather than a comprehensive enzyme-specific evaluation. This study aims to demonstrate the potential of thermosonication to improve the functional properties of poppy vinegar through a combination of experimental analyses, modeling, and computational approaches.

## 2 Materials and methods

### 2.1 Preparation of poppy vinegar

Poppy vinegar was produced using naturally grown, wild-harvested (fresh) poppy flowers (*Papaver rhoeas* L.) harvested during the spring season collected from the fields in Tekirdağ, Türkiye a region characterized by a temperate climate that naturally supports the widespread growth of this species. Prior to processing, visually deteriorated or darkened portions of the flowers were manually discarded to ensure raw material quality. Subsequently, the selected flowers were thoroughly cleaned and washed and vinegar production was carried out in accordance with the conventional two-stage fermentation procedure (24). This conventional method involves a primary alcoholic fermentation phase to convert available sugars into ethanol, sequentially followed by a secondary acetic acid fermentation phase where the produced ethanol is oxidized into acetic acid. Alcoholic fermentation was initiated by inoculating the substrate with *Saccharomyces cerevisiae* at an initial concentration of 10^6^ CFU/mL. Upon completion of ethanol production, acetic acid fermentation was induced by the addition of 5% (v/v) previously produced traditional organic apple vinegar from a previous batch as a starter culture. The fermentation process was conducted at 28 °C and allowed to proceed for up to 65 days under controlled conditions. During this period, the environment was kept under controlled conditions regarding light (dark), oxygen supply (cheesecloth). At the end of fermentation, total acidity reached approximately 4%, while residual ethanol levels ranged between 0.5 and 1%. The formation of a cellulose-rich biofilm (vinegar mother) was observed on the surface of the poppy vinegar, indicating active acetic acid bacterial metabolism. Following completion of fermentation, samples were transferred into sterile glass containers and preserved at −18 ± 1 °C until further analysis.

### 2.2 Thermosonication processing

A 100 mL poppy vinegar sample was subjected to the thermosonication-treated (TS) process, which was carried out using an ultrasonic device (Hielscher Ultrasonics Model UP200St, Berlin, Germany). The device operated at 26 kHz and 200 W. The ultrasound parameters evaluated within the scope of the research were amplitude (*X*₂, 60–100%), treatment time (*X*₁, 8–16 min), and temperature (*X*₃, 40–60 °C) values applied in constant mode. An ice bath was used to cool the samples. This was intended to prevent overheating during ultrasonic treatment. Samples stored at −18 ± 1 °C until analysis.

### 2.3 Thermal pasteurization

The poppy vinegar samples were pasteurised at 85 °C for 2 min in a water bath using the following equipment (Wisd model WUC-D06H, Daihan, Korea). Pasteurized (PAS) vinegar samples were cooled to 20 ± 1 °C and stored at −18 ± 1 °C until required.

### 2.4 Experimental design and data set

In this study, a three-factor Box–Behnken design (BBD) was employed to quantitatively evaluate the effects of thermosonication conditions on enzyme inhibitory activity. Experimental design methodologies are widely used to systematically investigate the influence of multiple variables and their interactions on response variables while minimizing the number of experimental runs. Among commonly applied designs, full factorial designs require a large number of experiments, whereas central composite designs (CCD) include axial points that extend beyond the experimental domain, potentially leading to impractical or unstable processing conditions in food systems. In contrast, BBD offers a more efficient and practical alternative by requiring fewer experimental runs and avoiding extreme factor combinations.

The independent variables were defined as treatment time (Time, min), amplitude (Amplitude, %), and temperature (Temperature, °C), while the dependent variables were α-glucosidase inhibition (%) and α-amylase inhibition (%). The design structure enabled the estimation of linear, quadratic, and interaction effects within the selected operating ranges, without requiring experiments at extreme levels. A total of 15 experimental conditions (*n* = 15) were generated, providing a balanced and efficient dataset suitable for response surface modeling and simultaneous evaluation of multiple responses. This design was therefore considered appropriate for capturing nonlinear process–response relationships while ensuring experimental feasibility and product stability under thermosonication conditions.

### 2.5 Machine learning models

This study used three different machine learning algorithms to model the effect of thermosonication on the inhibitory activity of α-glucosidase and α-amylase. The ability to produce reliable results on datasets with small sample sizes (*n* = 15) was the primary criterion for selecting the algorithm.

#### 2.5.1 Ridge regression

The problem of multicollinearity in multiple linear regression was solved by Hoerl and Kennard (25) with the proposal of a regularisation technique known as Ridge regression. This method limits the magnitude of model coefficients and prevents overfitting by adding an L2 penalty term to the loss function. The cost function of Ridge regression is calculated as shown in Equation 1.

$$J(B)=\sum {\left(y_{i}-{\hat{y}}_{i}\right)}^{2}+\alpha \sum {\beta_{j}}^{2}$$ (1)

In Equation 1, the term *α* is the regularization parameter and controls the balance between model complexity and goodness of fit. In this study, *α* = 0.1 for α-glucosidase prediction and *α* = 1.0 for α-amylase prediction were determined using hyperparameter optimization. To capture nonlinear relationships, a second-order polynomial transformation was applied to the input variables. As a result of this transformation, 9 features (3 main effects + 3 quadratic effects + 3 interactions) were derived from the 3 original variables.

#### 2.5.2 Support vector regression (SVR)

Support vector regression (SVR) is a type of machine learning algorithm that was developed by Vapnik (26) to solve regression problems. It is similar to support vector machines, but it has been adapted for use in regression tasks. SVR can model nonlinear relationships by mapping data to a higher-dimensional space. In this study, a radial basis function (RBF) kernel was used, and the hyperparameters were optimized as *C* = 1,000, *γ* = 0.01, and *ε* = 0.01–0.1.

#### 2.5.3 Bayesian Ridge regression

Bayesian Ridge regression provides a probability-based interpretation of classical Ridge regression (27). In this approach, rather than being fixed, the regularization parameter is automatically estimated from the data (28). It was preferred for providing uncertainty estimates in small datasets.

#### 2.5.4 Data preprocessing and pipeline structure

In machine learning models, data leakage can cause the model to learn from test data, leading to unrealistically high performance metrics. To prevent this problem, all data preprocessing steps are defined within the Pipeline structure of the scikit-learn library (29). Thanks to the Pipeline structure:

- *Z*-score normalization with StandardScaler
- Polynomial feature derivation with PolynomialFeatures
- Model training

Steps are performed only on the training data in each cross-validation iteration. This approach ensures that the test data is not used during the preprocessing phase.

#### 2.5.5 Hyperparameter optimization

In this study, the most suitable hyperparameter values for each algorithm were determined using leave-one-out cross-validation (LOOCV). The parameter combination yielding the highest LOOCV *R*^2^ was selected for each target variable. Table 1 shows the hyperparameter values obtained for each machine learning model. All modelling operations were performed using the Python 3.x language and the scikit-learn library (version 1.x) (29).

**Table 1:** Hyperparameter values of the machine learning models used.

Model	Parameter	α-glucosidase	α-amylase
Ridge (Poly2)	*α*	0.1	1.0
SVR (RBF)	*C*	1,000	1,000
SVR (RBF)	*γ*	0.01	0.01
SVR (RBF)	*ε*	0.01	0.1
Bayesian Ridge	–	Automatic	Automatic

Poly2, Ridge regression; SVR, support vector regression; RBF, radial basis function.

### 2.6 Model evaluation

#### 2.6.1 Cross-validation strategy

Accurate assessment of model performance in studies with limited sample sizes is of paramount importance to ensure robustness and generalizability. In the present investigation, model validation was performed using the leave-one-out cross-validation (LOOCV) approach based on a dataset comprising 15 observations. Within this validation framework, a single sample is iteratively excluded from the dataset and used as the test instance, while the remaining *n*−1 samples are employed for model training. This procedure is repeated n times so that each observation serves once as an independent test case (30). This approach minimizes variance in small samples, yielding more reliable performance predictions. In addition, 5-fold cross-validation was applied for comparison. However, high variance was observed in the 5-fold CV results because there were only 3 test samples in each fold in the 15-sample dataset. Therefore, LOOCV results were used to select the final model.

#### 2.6.2 Performance metrics

The performance of the machine learning models used in this study was evaluated using the following metrics:

- Coefficient of Determination (*R*^2^): It expresses the proportion of variance explained by the model and takes values between 0 and 1. An *R*^2^ value closer to 1 indicates greater predictive power. The mathematical formula for this metric is given in Equation 2.

$$R^{2}=1-\frac{\sum {\left(y_{i}-{\hat{y}}_{i}\right)}^{2}}{\sum {\left(y_{i}-{\bar{y}}_{i}\right)}^{2}}$$ (2)

- Root Mean Square Error (RMSE): Expresses the magnitude of the forecasting errors in the original unit. The mathematical formula for calculating the RMSE is shown in Equation 3.

$$\text{RMSE}=\sqrt{\sum {\left(y_{i}-{\hat{y}}_{i}\right)}^{2}/n}$$ (3)

- Mean Absolute Error (MAE): The MAE metric, whose mathematical formula is shown in Equation 4, is the average of the absolute values of the forecast errors and is less sensitive to outliers than RMSE.

$$\mathrm{MAE}=\sum |(y_{i}-{\hat{y}}_{i})|/n$$ (4)

- Mean Absolute Percentage Error (MAPE): The MAPE metric allows for comparisons between variables of different scales by expressing errors as percentages. The mathematical formula for the MAPE metric is shown in Equation 5.

$$\text{MAPE}=(100/n)\times \sum |(y_{i}-{\hat{y}}_{i})|/|y_{i}|$$ (5)

#### 2.6.3 Overfitting control

The model is said to be overfitted if it memorises the training data but is unable to generalise to new data. In this study, the risk of overfitting was controlled in two ways:

- The difference between the training *R*^2^ value and the LOOCV *R*^2^ value was calculated using Train-Test Gap Analysis. A difference below 0.15 was considered an indication that the model did not exhibit overfitting.
- Regularization of the L2 penalty term of the Ridge regression reduced the risk of overfitting by preventing excessive growth of model coefficients.

#### 2.6.4 Statistical significance test (permutation test)

In this study, a permutation test was used to assess whether the model’s performance was statistically significant. In the test, the target variable (*y*) values were first randomly mixed to eliminate the true relationship. Subsequently, the model was trained on the mixed dataset, and the LOOCV *R*^2^ was computed. This process was repeated 1,000 times to obtain the permutation *R*^2^ distribution. The *p*-value was calculated according to the position of the true *R*^2^ value in the permutation distribution. A *p*-value below 0.05 indicates that the model learned a true relationship, not a random one. Table 2 presents the evaluation metrics used in this study and the accepted ideal values for each metric.

**Table 2:** Summary of model evaluation metrics.

Metric	Explanation	Ideal value
*R* ^2^	Explained variance ratio	→ 1
RMSE	Estimation error (original unit)	→ 0
MAE	Mean absolute error	→ 0
MAPE	Percentage error	→ 0
Train-test gap	Indicator of overfitting	<0.15
*p*-Value	Statistical significance	<0.05

*R*^2^: coefficient of determination; RMSE, root mean square error; MAE, mean absolute error; MAPE, mean absolute percentage error.

#### 2.6.5 Uncertainty estimation of model performance

Due to the limited sample size (*n* = 15), additional uncertainty quantification was performed to assess the robustness of performance metrics. Bootstrap resampling (10,000 iterations) was applied to LOOCV prediction errors to estimate 95% confidence intervals for *R*^2^, RMSE, and MAE values. This approach provides distribution-based uncertainty bounds for performance metrics and reduces the risk of overinterpreting point estimates in small datasets.

### 2.7 Multiple target optimization

Multiple-target optimization was performed to determine the optimal thermosonication conditions that simultaneously maximize inhibition of α-glucosidase and α-amylase. For this purpose, three global optimization algorithms and three target functions were employed. The optimization algorithms used are described below in order:

- Differential Evolution (DE): Developed by Storn and Price (31), differential evolution is a population-based stochastic optimization algorithm. The algorithm converges to the global optimum using mutation, crossover, and selection operators. The main advantages of DE include the absence of gradient information, a small number of control parameters, and suitability for parallel computation. For this study, the population size was set at 15, the mutation factor was set within the range of 0.5–1.0, and the crossover probability was set at 0.7. The maximum number of iterations was set to 1,000.
- Basin Hopping (BH): This is a hybrid algorithm developed for global optimization. It can avoid local minima on energy surfaces by combining local search with stochastic perturbations. In this study, the L-BFGS-B algorithm was used for local search, and 100 iterations were performed.
- Dual Annealing (DA): This is an improved version of the classical simulated annealing algorithm. It combines both local and global search capabilities. It is effective in high-dimensional and multimodal optimization problems. The maximum number of iterations is set to 1,000.

Correlation analysis revealed a strong positive association between α-glucosidase and α-amylase inhibition responses (Pearson *r* = 0.74), indicating partially aligned objective behavior within the explored experimental domain. Consequently, the optimization problem did not involve a highly antagonistic trade-off. The convergence of different global optimization algorithms (DE, BH, DA) and aggregation strategies toward similar optimal conditions therefore reflects objective alignment and response surface smoothness rather than methodological redundancy. These findings suggest that the multi-objective framework primarily confirmed stability and consistency of optimal conditions rather than resolving strongly conflicting objectives.

#### 2.7.1 Objective functions

In multi-objective optimization, a set of optimal solutions is typically represented by a Pareto front, which captures the trade-offs among competing objectives rather than a single optimal point. In this study, three different approaches were employed to simultaneously maximize both inhibition responses. The first approach is based on the sum function, where the combined value of the two responses is maximized (Equation 6).

$$f(X)=Y_{1}(X)+Y_{2}(X)$$ (6)

The second approach is based on the weighted sum method, in which different levels of importance are assigned to each objective to achieve a balanced optimization outcome (Equation 7).

$$f(X)=W_{1}\times Y_{1}\mathrm{norm}(X)+W_{2}\times Y_{2}\mathrm{norm}(X)$$ (7)

The third approach is based on the desirability function, in which each response is transformed into a dimensionless desirability value, and an overall desirability index is calculated to simultaneously optimize multiple objectives (Equation 8).

$$D=\sqrt{\left(d_{1}\times d_{2}\right)}$$ (8)

#### 2.7.2 Optimization rules

The optimization processes carried out within the scope of this study were performed within the variable ranges determined in the experimental design:

- Duration (*X*₁): 8–16 min
- Amplitude (*X*₂): 60–100%
- Temperature (*X*₃): 40–60 °C

All optimization calculations were performed in the Python environment using the SciPy library (version 1.x). Table 3 shows the optimization algorithms and parameter values used in the study.

**Table 3:** Optimization algorithms and parameters.

Algorithm	Parameter	Value
Differential evolution	Population size	15
	Mutation factor	0.5–1.0
	Crossover probability	0.7
	Maximum iteration	1,000
Basin hopping	Local search	L-BFGS-B
	Number of iterations	100
Dual annealing	Maximum iteration	1,000

### 2.8 Analysis of phenolic compounds (HPLC)

Separation of the analytes was achieved on an ACE Genix C18 column (250 × 4.6 mm, 5 μm; Agilent Technologies) using established chromatographic conditions (32). The profiling of phenolic compounds was carried out using an Agilent 1,260 HPLC–DAD chromatographic system under gradient elution conditions. Analyses were performed at 30 °C with a fixed flow rate of 0.80 mL/min. The mobile phase consisted of solvent A (water containing 0.1% phosphoric acid, v/v) and solvent B, delivered according to a predefined gradient program. An injection volume of 10 μL was introduced for each run, and chromatograms were monitored at wavelengths of 280, 320, and 360 nm to ensure selective detection of phenolic constituents. Quantification of individual compounds was achieved based on calibration data, and results were expressed as μg/mL. Representative chromatograms and a brief method-validation summary (LOD/LOQ, recovery, repeatability) are provided in the Supplementary Figure S1.

### 2.9 Antidiabetic activity

The inhibitory activities of poppy vinegar against α-glucosidase and α-amylase were determined to assess its antidiabetic potential, employing a modified enzymatic protocol (33). Spectrophotometric readings were performed using an SP-UV/VIS-300SRB UV–Vis Spectrophotometer, and acarbose was included as a standard reference inhibitor for comparison in the antidiabetic analyses. The inhibitory activities of both enzymes were expressed as percentage inhibition (%) relative to the control, calculated as the reduction in absorbance relative to the enzyme activity in the absence of the sample.

### 2.10 Bioactive compounds

Total phenolic compounds (TPC) were quantified by the Folin–Ciocalteu method based on the reduction of the reagent by phenolic constituents in methanolic extracts. After centrifugation, the supernatants were mixed with Folin–Ciocalteu reagent and sodium carbonate, incubated in the absence of light, and absorbance was recorded at 765 nm (34). The TPC was calculated from a gallic acid calibration curve and expressed in terms of mg GAE per 100 mL of the sample. Total flavonoid content (TFC) was quantified through a colorimetric assay involving the complexation of flavonoids with aluminum chloride under controlled conditions (35). For TFC determination, diluted poppy vinegar extracts were subjected to a sequential reaction with NaNO₂, AlCl₃, and NaOH to form flavonoid–aluminum complexes. Absorbance values were recorded at 510 nm, and quantification was performed using a calibration curve prepared with catechin as the reference standard. The findings were expressed as milligrams of catechin equivalents per liter (mg CE/L). All analyses were conducted in triplicate to enhance methodological precision and reproducibility.

The total anthocyanin content (TAC) was assessed using the pH differential assay, which is based on the structural transformation of anthocyanins under different pH conditions (36, 37). Poppy vinegar samples were diluted (1:10, v/v), vortex-mixed, and centrifuged (4,000 rpm, 10 min) to obtain a clarified aqueous extract. For TAC analysis, aliquots of the extract were separately adjusted to 5 mL with potassium chloride buffer (0.025 M, pH 1.0) and sodium acetate buffer (0.4 M, pH 4.5). After equilibration at ambient temperature for 15–30 min, absorbance values were measured at 515 and 700 nm using an SP-UV/VIS-300SRB UV–Vis Spectrophotometer against a distilled water blank. The total anthocyanin content was calculated and expressed as milligrams of cyanidin-3-glucoside equivalents per 100 mL of the sample (mg C3GE/100 mL).

### 2.11 Biological and antioxidant activity

Antioxidant capacity was further evaluated by determining free radical scavenging potential through the 2,2-diphenyl-1-picrylhydrazyl (DPPH•) assay. This spectrophotometric method is based on the reduction of the stable DPPH• radical by antioxidant molecules, resulting in a measurable decrease in absorbance (38). Equal volumes (1 mL) of the sample and 0.2 mM methanolic DPPH• solution were mixed and incubated for 30 min at 25 ± 1 °C in the absence of light. The decrease in absorbance was then monitored at 517 nm using an SP-UV/VIS-300SRB UV–Vis Spectrophotometer. The free radical scavenging activity was calculated using a Trolox standard calibration curve, and the final results were expressed as milligrams of trolox equivalent antioxidant capacity per milliliter of the sample (mg TEAC/mL).

### 2.12 Molecular docking study

Molecular docking studies were performed using AutoDock Vina 1.2.7 to model the binding of the compounds to α-amylase, a crucial target for diabetes (39). The compounds were selected based on analytical results that highlight variations in phenolic content attributable to distinct vinegar production processes. The 3-D crystal structure of the selected target protein, alpha-amylase, was obtained from the RCSB Protein Data Bank as entry 1HNY (40). The α-amylase structure was initially refined using UCSF Chimera 1.19 by removing water molecules, salt ions, and other heteroatoms (41). Following this preparation, the optimal binding conformation was determined as the pose with the highest binding affinity score.

For α-amylase, the grid box was centered at *X*: 10.0, *Y*: 50.0, *Z*: 18.0 with box size *X* = 20; *Y* = 20; *Z* = 20. The active site, comprising the catalytic triad amino acids D197, E233, and D300, is included in the grid box. Docking was run using Vina 1.2.7, and results were visualized with UCSF Chimera 1.19. The 3D SDF structure of quercetin was obtained from PubChem (42). 3-D SDF structure of gallic acid was obtained from PubChem (43). 3-D SDF structure of trans-cinnamic acid was obtained from PubChem (44). Docking studies were run using Vina 1.2.7, and Biovia Discovery Studio was used for visualization and binding analysis (45). Model validation was performed by redocking the cognate ligand, acarbose, to the α-amylase receptor protein. Acarbose obtained from 1B2Y crystal and aligned with 1HNY with RMSD 0.131 Å (Supplementary Figure S2), and re-docking was performed to the gridbox of the receptor 1B2Y, placing in the same space coordinates as 1HNY (Supplementary Figure S3). An RMSD of less than 2 Å (0.650 Å) for acarbose validated our protocol (Supplementary Figure S4) (46).

### 2.13 Statistical analysis

All measurements were performed using independent biological replicates, and each biological replicate was analyzed in technical triplicate; results are reported as mean ± SD. Biological replicates (*n* = 3 per group) were defined as independently prepared/processed vinegar batches (independent runs) for each treatment group (CON, TS, and PAS), whereas technical replicates (*n* = 3) refer to repeated analytical measurements (e.g., replicate assay reads or HPLC injections) performed on the same prepared sample/extract and were used to assess analytical precision only. Group differences among untreated control (CON), thermosonication-treated (TS), and pasteurized (PAS) samples were assessed by one-way analysis of variance (ANOVA) followed by Tukey’s *post hoc* multiple comparison test, with statistical significance set at *p* < 0.05. Statistical analyses were conducted using GraphPad Prism and SPSS (version 20.0; SPSS Inc., Chicago, USA). For machine-learning modeling, all computations were performed in Python (scikit-learn), and model performance was primarily evaluated using leave-one-out cross-validation (LOOCV) due to the limited sample size (*n* = 15). Predictive accuracy was quantified using the coefficient of determination (*R*^2^), RMSE, MAE, and MAPE; additionally, the statistical significance of model performance was assessed using a permutation test (1,000 permutations), with *p* < 0.05 considered significant.

## 3 Result and discussion

### 3.1 Model performance comparison

This study compared the predictive performance of three machine learning algorithms for estimating α-glucosidase and α-amylase inhibition. The performance metrics obtained using leave-one-out cross-validation (LOOCV) are presented in Table 4.

**Table 4:** Comparison of LOOCV performance of machine learning models.

Model	Aim	*R* ^2^	RMSE	MAE	MAPE (%)
Ridge (Poly2)	α-glucosidase	0.9414	0.237	0.166	0.40
SVR (RBF)	α-glucosidase	0.9340	0.252	0.207	0.50
Bayesian Ridge	α-glucosidase	0.9405	0.239	0.167	0.41
Ridge (Poly2)	α-amylase	0.7951	0.362	0.305	0.83
SVR (RBF)	α-amylase	0.7830	0.373	0.294	0.80
Bayesian Ridge	α-amylase	0.7712	0.383	0.311	0.84

*R*^2^: coefficient of determination; RMSE, root mean square error; MAE, mean absolute error; MAPE, mean absolute percentage error; SVR, support vector regression; RBF, radial basis function kernel; Poly, polynomial features (used with Ridge Regression).

For α-glucosidase inhibition, Ridge regression (Poly2) achieved the highest predictive performance, with an LOOCV *R*^2^ of 0.9414, indicating that approximately 94% of the variance in the observed data was explained by the model. The SVR and Bayesian Ridge models also exhibited similarly high performance, with *R*^2^ values of 0.9340 and 0.9405, respectively. In terms of prediction error, Ridge regression yielded the lowest RMSE value (0.237), confirming its superior accuracy.

In contrast, model performance for α-amylase inhibition was comparatively lower. Although Ridge regression still provided the best predictive performance (LOOCV *R*^2^ = 0.7951), it did not reach the level observed for α-glucosidase inhibition. This discrepancy suggests that α-amylase inhibition may be governed by more complex and nonlinear relationships with the processing parameters.

Analysis of MAPE values further supported the robustness of the models, with prediction errors of 0.40% for α-glucosidase and 0.83% for α-amylase, both of which fall within acceptable limits for practical applications.

Bootstrap-derived 95% confidence intervals provided additional support for the stability of the LOOCV performance metrics. For α-glucosidase, the 95% confidence interval for *R*^2^ remained relatively narrow, indicating a robust explanation of variance despite the limited sample size. In contrast, the confidence intervals for α-amylase were moderately wider, suggesting greater sensitivity of the model to sample variability. These results indicate that the reported performance metrics are reliable, although they should be interpreted within the associated uncertainty bounds.

### 3.2 Model validation

#### 3.2.1 Experimental and predicted values

**Figure 1:**
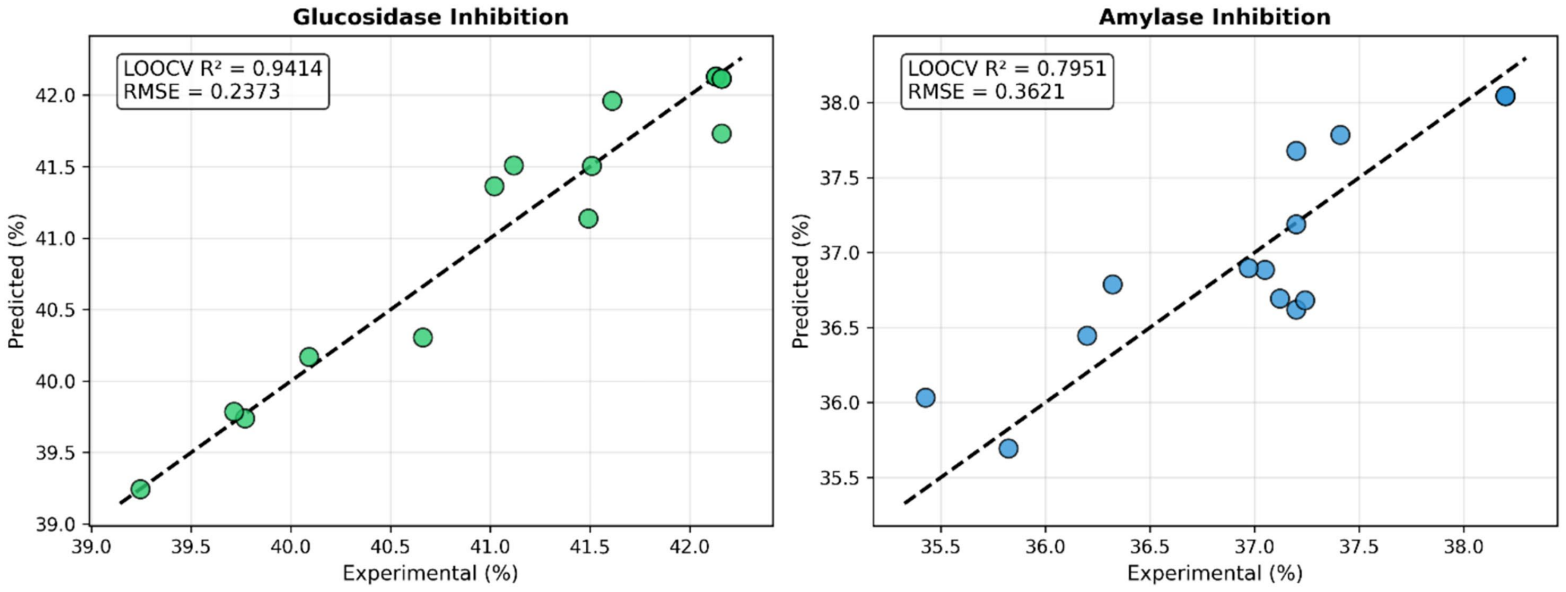
Ridge regression displays actual and predicted values.

Figure 1 shows the relationship between experimental and predicted values for Ridge regression (Poly2). For both target variables, the data points are close to the 1:1 line, indicating the model’s successful predictive performance. While the distribution of points is tighter for α-glucosidase, a relatively greater deviation is observed for α-amylase.

**Figure 2:**
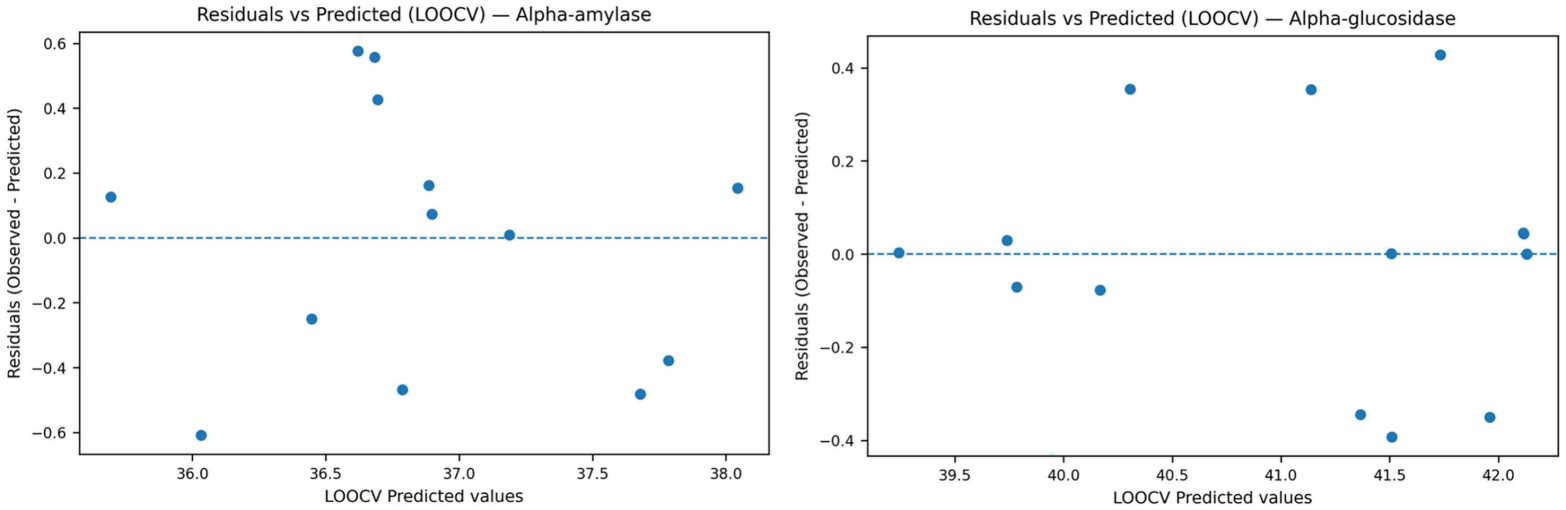
Residuals versus predicted values (LOOCV) for α-glucosidase and α-amylase inhibition models.

Residual diagnostics were performed using LOOCV predictions to evaluate potential systematic bias. As shown in Figure 2, residuals were randomly dispersed around zero without observable trends, curvature, or funnel-shaped patterns. No heteroscedasticity or structural bias was detected for either response variable. These findings support adequacy of the second-order polynomial Ridge regression model within the defined experimental domain.

#### 3.2.2 Overfitting analysis

To assess whether the model exhibited overfitting, training and test performances were compared. The Train-Test gap for the α-Glucosidase model was calculated as 0.055, well below the acceptable limit of 0.15. For the α-Amylase model, the gap was 0.185, slightly above the limit, but thanks to Ridge regularization, while the Train–Test gap for α-amylase (0.185) slightly exceeded the predefined threshold (0.15), Ridge regularization mitigated coefficient instability; however, this result indicates moderate variance sensitivity and warrants cautious interpretation.

#### 3.2.3 Permutation test results

The statistical significance of model performance was evaluated using a permutation test. A *p*-value <0.01 was obtained for both target variables, confirming that the models learned real patterns instead of random relationships. Table 5 shows the permutation test results.

**Table 5:** Permutation test results (*n* = 1,000 permutations).

Aim	Real *R*^2^	Permutation *R*^2^ (mean ± std)	*p*-Value	Result
α-glucosidase	0.9414	−2.71 ± 2.28	0.001	Significant
α-amylase	0.7951	−2.09 ± 1.83	0.003	Significant

### 3.3 Optimization results

#### 3.3.1 Algorithm comparison

In this study, 9 optimization studies were conducted using three optimization algorithms (DE, BH, DA) and three objective functions. Table 6 presents the results obtained for all combinations.

**Table 6:** Comparison of optimization algorithms.

Method	Time (min)	Amplitude (%)	Temp (°C)	α-glucosidase (%)	α-amylase (%)	Total (%)
DE-sum	13.86	84.68	47.40	42.34	37.96	80.30
BH-sum	13.86	84.68	42.34	37.96	37.96	80.30
DA-sum	13.86	84.68	42.34	37.96	37.96	80.30
DE-weighted	13.68	84.37	47.62	42.32	37.98	80.30
BH-weighted	13.68	84.37	47.62	42.32	37.98	80.30
DA-weighted	13.68	84.37	47.62	42.32	37.98	80.30
DE-desirability	13.31	83.77	48.04	42.28	38.02	80.29

The highest value in terms of total inhibition (α-glucosidase + α-amylase) was obtained with the total function of the Differential Evolution algorithm (80.30%). It was observed that all algorithms converged to similar optimal points, supporting the reliability of the results.

#### 3.3.2 Optimal conditions

Under the determined optimal thermosonication conditions (13.86 min, 84.68% amplitude, 47.40 °C), the model predicted α-glucosidase inhibition of 42.34% and α-amylase inhibition of 37.96%. The validation experiment was conducted under the same conditions, yielding 41.73 and 36.11%, respectively. The closeness of the predicted and experimental values indicates that the operating point defined in the optimization process is reproducible and that the model captures the process-response relationship with sufficient accuracy. Indeed, the fact that the prediction-experiment difference for α-glucosidase inhibition remains at only 0.61 percentage points (≈1.44% relative deviation) suggests that the optimum region is captured with a narrow uncertainty band for this response, and that even small fluctuations in process parameters have a limited impact on efficiency. In contrast, the difference in α-amylase inhibition being 1.85 percentage points (≈4.87% relative deviation) suggests that this response may be more sensitive to factors or that the response surface exhibits a more complex (non-linear/interaction-weighted) character; furthermore, matrix-dependent variability in enzyme inhibition analyses (such as intraday fluctuations in enzyme activity, substrate concentration, reaction time, and temperature control) can increase the deviation in α-amylase measurements. However, the fact that both inhibitor efficacies were achieved at levels close to the expected values in the validation experiment indicates that the selected optimal conditions achieve the aim of simultaneously enhancing both targets. Consequently, the combination of 13.86 min–84.68%–47.40 °C provides a reliable operational window that can be used in the scaling-up and product development phases of the process, both for producing validation outputs consistent with model-based predictions and for optimizing the inhibition of both enzymes.

#### 3.3.3 Response surface analysis

**Figure 3:**
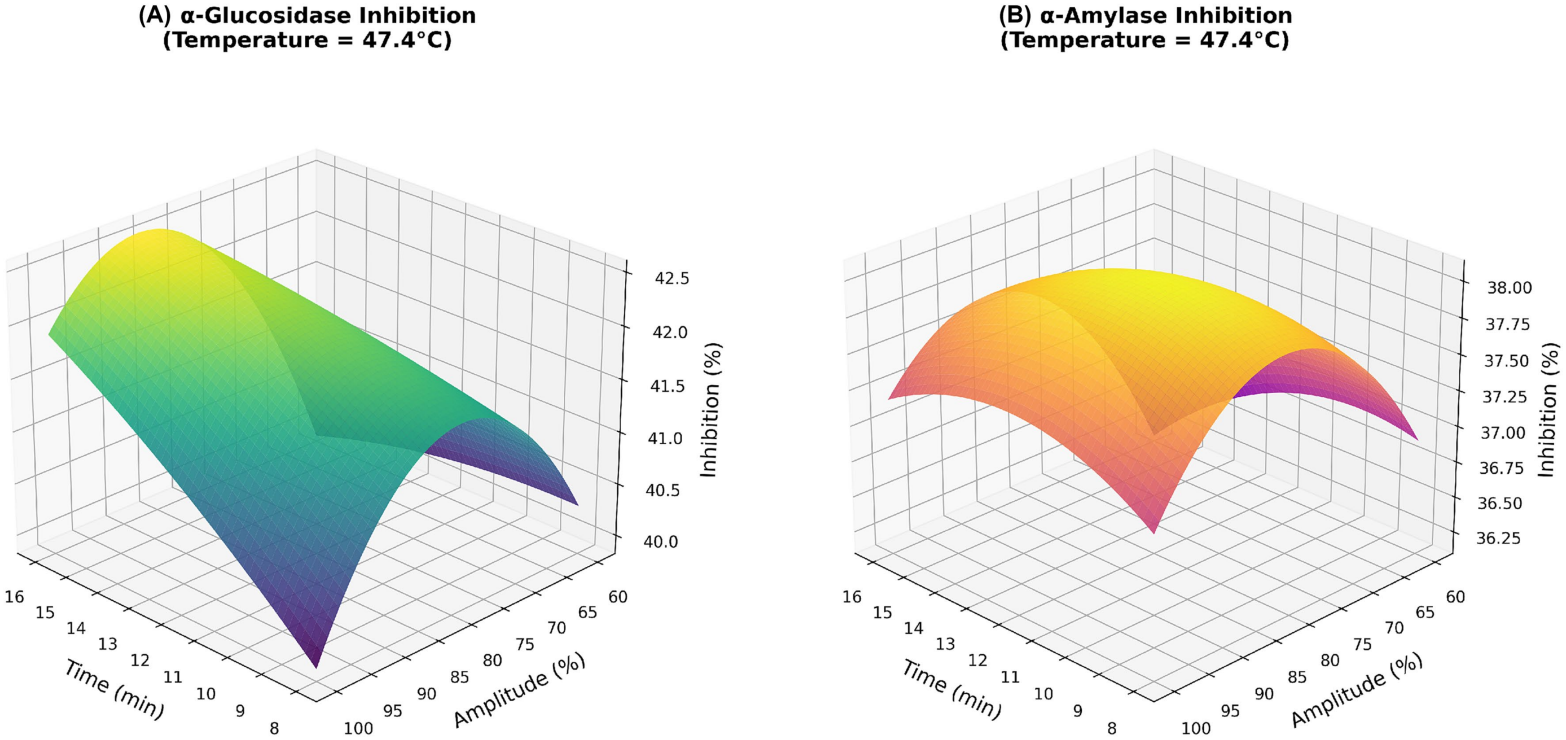
3D response surface plots showing the interactive effects of extraction time and amplitude on **(A)** α-glucosidase inhibition and **(B)** α-amylase inhibition.

**Figure 4:**
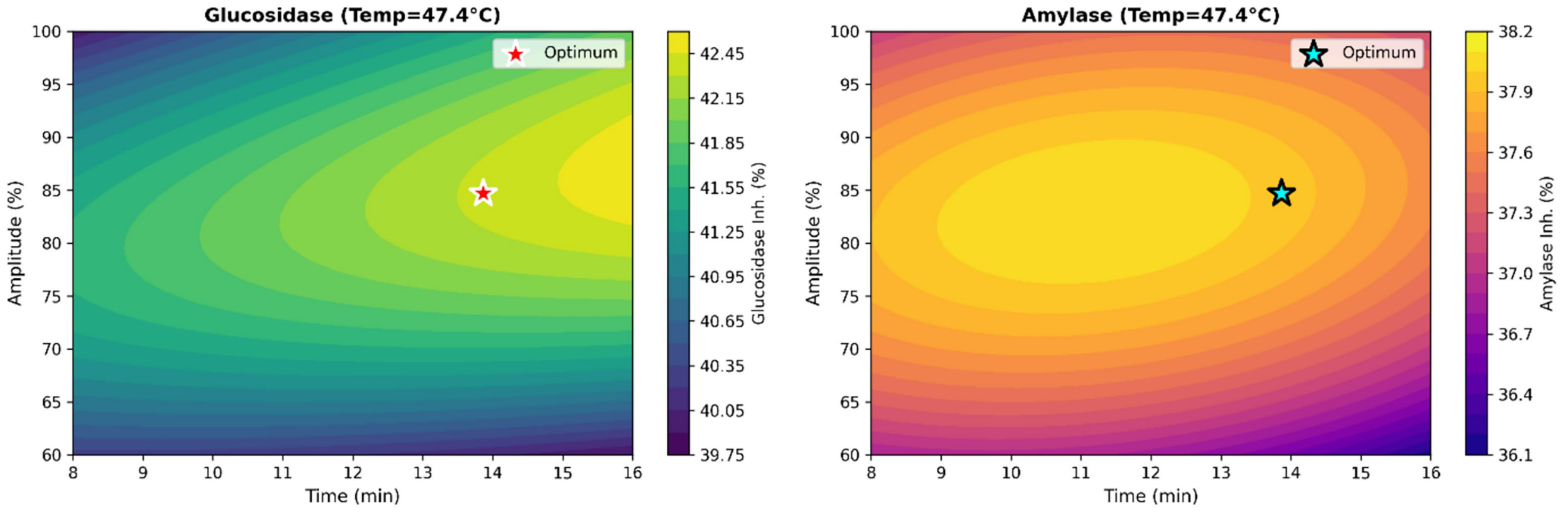
Contour graphs.

Figures 3, 4 present the 3D response surface and corresponding contour plots, respectively. A distinct optimal region is observed for both response variables. The optimum point, indicated by the red star in the contour plots, lies in the central region where the highest inhibition values are observed.

Based on the response surface plots showing the interaction between amplitude and treatment time at a fixed temperature, both parameters positively affect enzyme inhibition over the studied range. Increasing amplitude increases acoustic cavitation intensity, thereby improving cell disruption and mass transfer and facilitating the release of bioactive compounds responsible for enzyme inhibition. Similarly, extending the treatment time promotes the diffusion of intracellular constituents into the liquid phase, thereby increasing inhibitory activity up to an optimal level.

Although temperature was held constant in the plots presented, its effect was evaluated using the overall model and complementary response surface analyses. The results indicate that moderate temperatures (45–50 °C) yield the highest inhibition responses, whereas lower or higher temperatures may reduce effectiveness due to insufficient extraction or thermal degradation of heat-sensitive bioactive compounds. These findings highlight the synergistic role of thermosonication parameters in modulating enzyme inhibition and are consistent with previous studies reporting enhanced bioactive release under controlled ultrasound-assisted processing conditions.

As demonstrated by this study, machine learning models can be successfully applied to datasets with small sample sizes (*n* = 15). Ridge regression (Poly2) exhibited the best performance for both target variables. This result once again confirms the effectiveness of L2 regularization, proposed by Hoerl and Kennard (25), in preventing overfitting in small datasets. It should be noted that the second-order polynomial transformation increased the predictor dimensionality to nine features (three linear, three quadratic, and three interaction terms) relative to 15 samples, corresponding to a feature-to-sample ratio of 0.6. Although this represents moderate dimensionality in a small dataset context, L2 regularization was specifically employed to constrain coefficient variance and reduce model instability. Therefore, model complexity was controlled through coefficient shrinkage rather than feature elimination.

Ridge regression was selected in this study for its ability to model nonlinear relationships with polynomial feature transformations, while simultaneously controlling model complexity through L2 regularization (*α*). This regularization mechanism constrains coefficient magnitudes, thereby reducing the risk of overfitting, which is particularly critical in small datasets. In addition, Ridge regression offers computational efficiency due to its closed-form solution, making it well-suited for iterative modeling and optimization tasks.

More complex models, including ensemble-based methods (e.g., Random Forest, XGBoost, Gradient Boosting) and deep learning approaches, were not employed in this study. This decision was primarily driven by the limited dataset size (*n* = 15), which is generally insufficient for such models to achieve reliable generalization. These methods typically require larger datasets to capture underlying patterns effectively; otherwise, they are prone to overfitting and unstable predictions.

The relatively low *R*^2^ value (0.795) observed for α-amylase inhibition may be attributed to several factors. First, the relationship between thermosonication parameters and α-amylase inhibition is likely more complex and may involve higher-order nonlinear interactions beyond second-order polynomial representation. Second, experimental variability and measurement uncertainty associated with α-amylase activity may have contributed to reduced model performance.

One of the important methodological contributions of this study is the measures taken to prevent data leakage. A common mistake in machine learning applications is to scale and perform feature engineering on the entire dataset. This leads to information leaking from the test data into the training process and unrealistically high performance metrics. In our study, this problem was solved using the scikit-learn Pipeline structure (29). The Pipeline ensures that the StandardScaler and PolynomialFeatures transformations are fitted only on the training data in each cross-validation iteration. The checks confirmed that the difference in *R*^2^ between the leaky and leak-free methods is negligible (0.0003). It is noteworthy that the three different global optimization algorithms (DE, BH, DA) used in the study converge to similar optimum points. This indicates that the obtained optimal point is reliable and that the response surface is relatively smooth. The Differential Evolution algorithm demonstrated the best performance in both convergence rate and final solution quality. As Storn and Price (31) stated, the DE algorithm is easy to use in practice because it has a small number of control parameters and does not require gradient information. These advantages of DE were also observed in this study.

**Figure 5:**
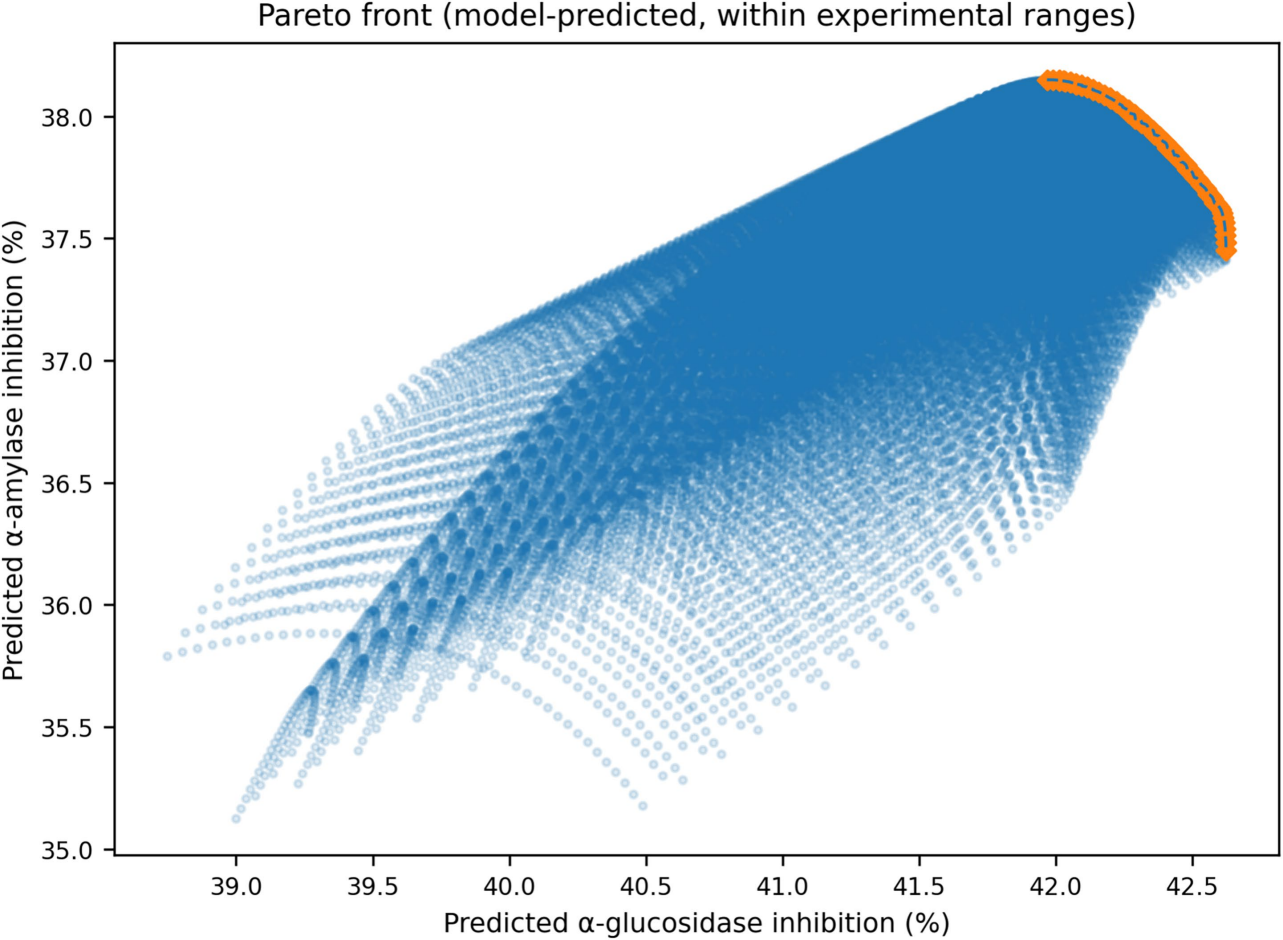
Pareto front illustrating trade-off structure between α-glucosidase and α-amylase inhibition responses.

The Pareto front constructed using model-predicted responses within the experimental design space is presented in Figure 5. The resulting frontier exhibits a relatively narrow, smooth structure, reflecting a partial alignment between the inhibition responses to α-glucosidase and α-amylase. While the objectives are not strictly identical, the trade-off region is limited, which explains the convergence of different global optimizers and aggregation strategies toward similar optimal conditions. This behavior indicates that the optimization problem does not involve a strongly antagonistic objective structure but rather partially aligned responses within the studied domain.

### 3.4 Phenolic compounds

Phenolic compounds constitute a diverse class of plant-derived secondary metabolites structurally defined by the presence of one or more hydroxyl substituents bound to an aromatic ring. These constituents range from relatively simple phenolic acids to highly complex polymeric structures. Beyond their biological relevance, phenolic compounds significantly contribute to the fundamental sensory attributes of vinegar, particularly influencing its characteristic color profile and flavor perception (47, 48). Table 7 reveals that the phenolic profile of poppy vinegar is dominated by a small number of compounds, with gallic acid as the dominant component (44.73–50.26 μg/mL). The researchers found Gallic acid levels of 49.19 ± 0.69 μg/mL in the CON group, 50.26 ± 4.34 μg/mL in the TS group, and 44.73 ± 2.64 μg/mL in the PAS group. They observed no significant difference between the treatments, as they shared the same superscript letter within the same row (*p* < 0.05). Similarly, protocatechuic acid (1.01–1.17 μg/mL), rutin (0.19–0.24 μg/mL), and neohesperidin (0.47–0.55 μg/mL) values were also maintained regardless of processing conditions (*p* < 0.05). These results show that the phenolic composition is clustered around a few dominant compounds, and that process-induced changes primarily shape the product’s phenolic signature through these main compounds. In the study by Yıkmış et al. (49), who treated poppy vinegar with ultrasound, the phenolic compound composition was also determined with the highest values of gallic acid 47.78 ± 0.32 and protocatechuic acid 1.21 ± 0.04. The compounds that changed significantly across processing treatments were quercetin and trans-cinnamic acid, indicating marked process sensitivity for these analytes (*p* < 0.05). Quercetin was detected only in the TS sample at 0.25 ± 0.02 μg/mL, while it remained below the detection limit in CON and PAS (reported as 0.00 ± 0.00 μg/mL; *p* < 0.05). This pattern suggests that thermosonication may modulate the release and/or partitioning of specific phenolic fractions, potentially by altering intra-matrix mass transfer and solubility behavior in a compound-dependent manner. Nevertheless, given the very low quercetin level relative to the major phenolics, and because compound-level association analyses (e.g., correlations/regressions with enzyme inhibition) and synergistic interaction assessments were not conducted, these findings should be interpreted as compositional shifts rather than direct evidence of causality for the observed bioactivity. Consistent with this interpretation, prior studies in fruit juices and related matrices have reported that ultrasound-assisted or combined mild processing can preserve—and in some cases increase—measured phenolic indices compared with conventional thermal pasteurization, with the direction and magnitude of change depending on processing intensity and matrix structure (50–52). Overall, the inhibition response is more appropriately viewed as matrix-driven rather than attributable to a single phenolic compound.

**Table 7:** Phenolic compound concentrations (μg/mL) in poppy vinegar subjected to different processing treatments (CON, TS, PAS).

Compound (μg/mL)	CON	TS	PAS
Gallic acid	49.19 ± 0.69^a^	50.26 ± 4.34^a^	44.73 ± 2.64^a^
Protocatechuic acid	1.07 ± 0.01^a^	1.17 ± 0.10^a^	1.01 ± 0.09^a^
Catechin	nd	nd	nd
Hydroxybenzoic acid	nd	nd	nd
Vanillic acid	nd	nd	nd
Gentisic acid	nd	nd	nd
*p*-coumaric acid	nd	nd	nd
Rutin	0.19 ± 0^a^	0.22 ± 0.02^a^	0.24 ± 0.02^a^
Ferulic acid	nd	nd	nd
Naringin	nd	nd	nd
*o*-coumaric acid	nd	nd	nd
Neohesperidin	0.55 ± 0.01^a^	0.55 ± 0.05^a^	0.47 ± 0.05^a^
Coumarin	nd	nd	nd
Resveratrol	0.01 ± 0.00^b^	0.00 ± 0.00^a^	0.01 ± 0.00^b^
Quercetin	0.00 ± 0.00^a^	0.25 ± 0.02^b^	0.00 ± 0.00^a^
*trans*-cinnamic acid	0.18 ± 0.00^b^	0.00 ± 0.00^a^	0.95 ± 0.07^c^
Hesperidin	nd	nd	nd
Alizarin	nd	nd	nd
Flavon	nd	nd	nd

Values are presented as mean ± SD. CON vinegar: control (untreated) vinegar; TS vinegar: thermosonication-treated vinegar; PAS vinegar: pasteurized vinegar. ND: not detected. Different superscript letters within the same row indicate statistically significant differences among treatments (*p* < 0.05).

In terms of trans-cinnamic acid, the highest value was observed with PAS: 0.95 ± 0.07 μg/mL, while CON: 0.18 ± 0.00 μg/mL was lower, and it decreased to 0.00 ± 0.00 μg/mL in TS (*p* < 0.05); this suggests that pasteurization may support the formation/release of this compound, while thermosonication may reduce its detectable level by accelerating its conversion or degradation processes. In contrast to our study, the level of trans-cinnamic acid in purple onion juice increased after thermosonication (53). Furthermore, resveratrol levels remained at 0.00 ± 0.00 μg/mL in TS, while they were 0.01 ± 0.00 μg/mL in CON and PAS, with TS standing out from the others (*p* < 0.05); therefore, it is understood that the process effect creates a more selective and pronounced response pattern, especially with low-concentration compounds.

### 3.5 Effects of processing on α-glucosidase and α-amylase inhibition

**Figure 6:**
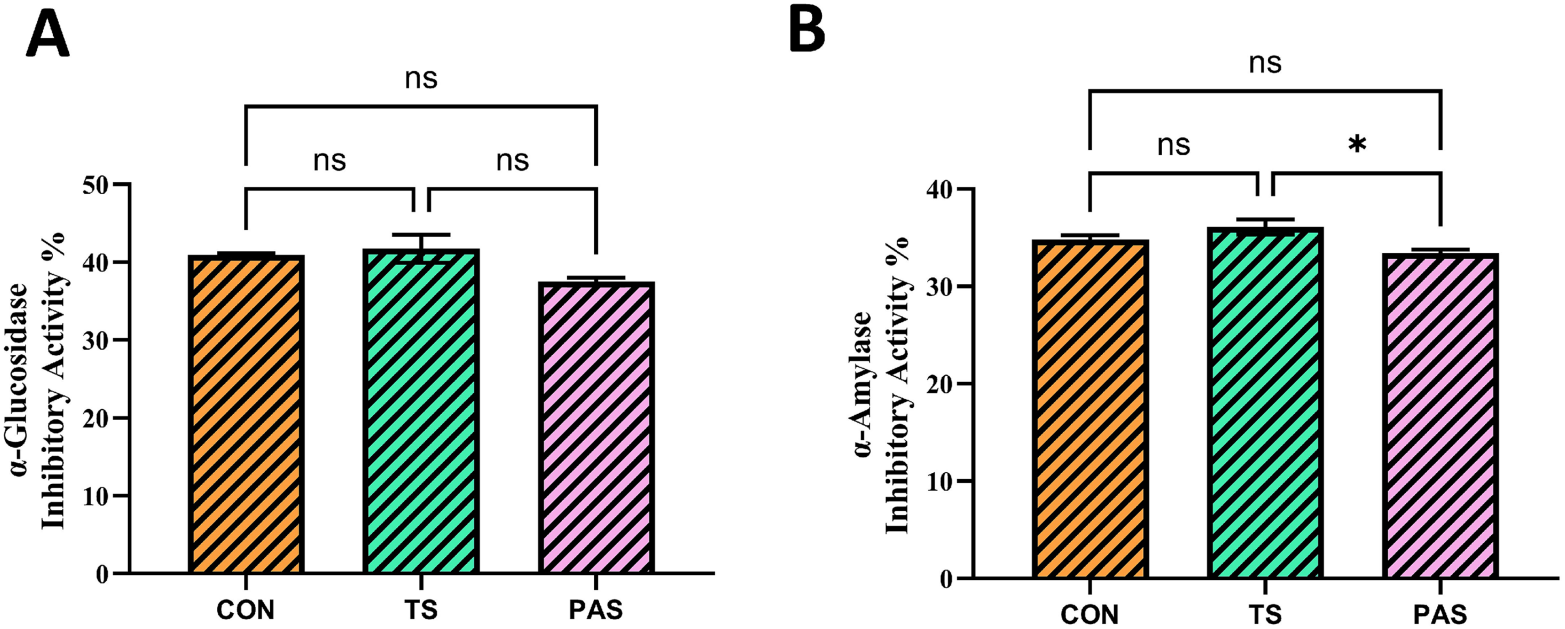
Antidiabetic inhibitory activity of vinegar samples under different processing treatments: **(A)** α-glucosidase inhibitory activity and **(B)** α-amylase inhibitory activity. CON vinegar: control (untreated) vinegar; TS vinegar: thermosonication-treated vinegar; PAS vinegar: pasteurized vinegar. ND: not detected. Values are presented as mean ± SD (*n* = 3). ^*^Indicates a statistically significant difference compared with CON vinegar (*p* < 0.05).

In Figure 6A, α-glucosidase inhibition is observed to be 40.94, 41.73, and 37.52% in the CON, TS, and PAS groups, respectively. No statistically significant difference (*p* > 0.05) was observed in the α-glucosidase inhibitory activity of poppy vinegar between untreated control samples and those subjected to thermosonication. A similar result was obtained with hawthorn vinegar after ultrasound treatment (54). The fact that TS exhibited a level very close to, and slightly higher than, that of the control group suggests that thermosonication at least preserved (or even provided a limited increase in) inhibitory capacity, whereas the lower value in the PAS group indicates that inhibitory activity may be weakened by heat treatment. Numerically, the increase in TS compared to CON was 0.79% (approximately 1.93% relative increase based on CON), while the PAS value was 4.21% lower than TS (approximately 10.09% relative decrease based on TS) and 3.42% lower than CON (approximately 8.35% relative decrease based on CON), quantitatively supporting the tendency of heat treatment to reduce inhibitory capacity. Another study with a similar design to ours found that the decrease in α-glucosidase inhibition in pasteurized purple onion juice (37.95%) was not statistically significant relative to the control group (40.45%) (53). If the figure contains significance markers/letter groups, it can be stated that this decrease is significant at *p* < 0.05, especially when PAS is separated from other groups; this pattern is consistent with a partial reduction in heat-sensitive phenolic fractions or synergistic component interactions during pasteurization. Furthermore, our findings regarding the antidiabetic potential of this functional vinegar align with the broader understanding of fermentation-induced bioactivity. Previous literature strongly highlights that fermentation processes inherently enhance the release and biotransformation of bioactive compounds. For instance, it has been demonstrated that other fermented products, such as fruit wines, exhibit robust α-glucosidase inhibitory activity, and, notably, this inhibitory activity is significantly greater in the fermented product than in its unfermented raw material (55).

Figure 6B shows the α-amylase inhibition ranking as TS (36.11%) > CON (34.83%) > PAS (33.415%), indicating that thermosonication has a process profile that better preserves inhibitory activity. The increase in TS compared to CON is 1.28% (approximately 3.67% relative increase based on CON), while the difference in TS compared to PAS is 2.695% (approximately 8.07% relative increase based on PAS); the difference in CON compared to PAS is calculated as 1.415% (approximately 4.06% relative decrease based on CON). These results suggest that thermosonication has the potential to preserve/improve inhibitory capacity in both enzyme targets, while pasteurization may partially limit the effectiveness of heat-sensitive inhibitory components. As in our study, the hawthorn vinegar sample treated with ultrasound exhibited statistically significantly higher α-amylase inhibitor activity than the pasteurized sample, as reported by Öğüt et al. (54). α-amylase is a principal digestive enzyme that hydrolyzes complex carbohydrates into simpler sugars, ultimately contributing to glucose release. Suppression of this enzymatic activity delays carbohydrate degradation and subsequent glucose absorption, which in turn may attenuate postprandial elevations in blood glucose concentrations (56, 57).

### 3.6 Impact of processing on antioxidant capacity and bioactive profile

**Figure 7:**
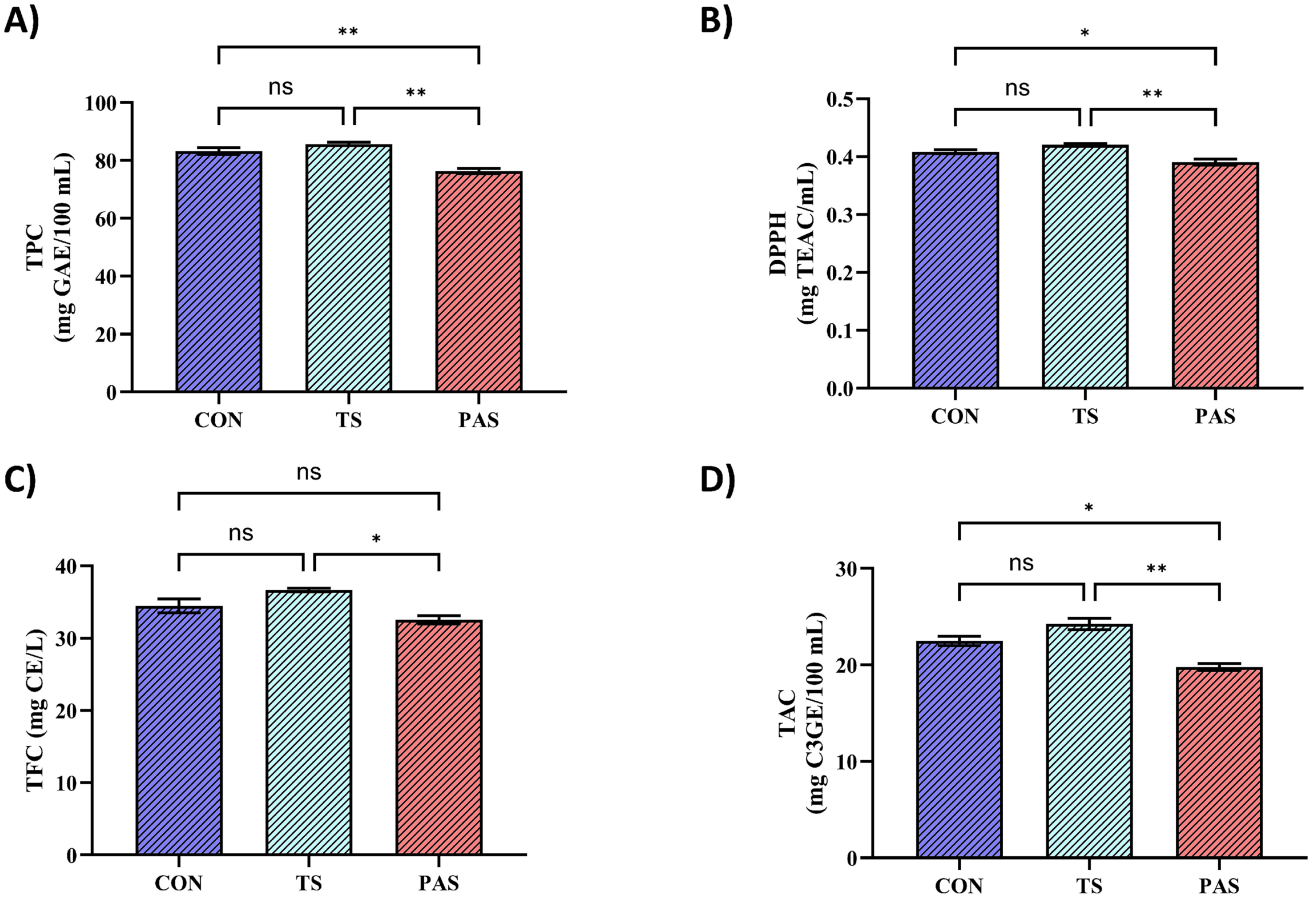
Effects of thermosonication and pasteurization on the bioactive profile and antioxidant capacity of vinegar samples: **(A)** total phenolic content (TPC), **(B)** DPPH radical scavenging activity, **(C)** total flavonoid content (TFC), and **(D)** total anthocyanin content (TAC) in CON (untreated), TS (thermosonication-treated), and PAS (pasteurized) vinegars. Values are presented as mean ± SD. ND, not detected. Asterisks denote significance levels where indicated: ^*^p < 0.05, ^**^p < 0.01.

In Figure 7A, the thermosonication (TS) group showed the highest TPC value (85.73 ± 0.56 mg GAE/100 mL), whereas the control (CON) group exhibited a very similar level (83.3 ± 1.18 mg GAE/100 mL); accordingly, no significant difference was observed between CON and TS (*p* > 0.05; ns). In contrast, the PAS group exhibited a lower TPC (76.32 ± 0.93 mg GAE/100 mL) that remained significantly lower than those of both CON and TS (*p* < 0.01). Notably, the absolute magnitude of the TS–PAS difference is modest (~9 mg GAE/100 mL); therefore, these results are interpreted primarily as indicating better retention of phenolic-related indices under TS than under pasteurization, rather than as a large enhancement. Similar trends have been reported in thermosonicated rocket juice, where pasteurization reduced TPC, whereas thermosonication yielded comparable or slightly higher values, depending on processing conditions (58). The increase observed in bioactive compound levels following thermosonication treatment is thought to be associated with the mechanical forces generated by ultrasonic cavitation, which disrupt cell walls and the structural integrity of the cellular matrix. This mechanical effect facilitates the release of intracellular constituents, thereby enhancing the extraction efficiency of phenolics and other secondary metabolites (20). In Figure 7B, the DPPH radical scavenging capacity exhibited a profile consistent with TPC values: TS 0.42 ± 0.00 mg TEAC/mL, CON 0.41 ± 0.00 mg TEAC/mL, and PAS 0.39 ± 0.00 mg TEAC/mL. Similarly, no significant difference was found between CON and TS (*p* > 0.05; ns), whereas the TS–PAS comparison indicated a significantly higher antioxidant capacity in favor of TS (*p* < 0.01). In addition, the lower value observed in PAS compared to CON was statistically significant (*p* < 0.05). Taken together, these findings suggest that thermosonication at least preserves antioxidant capacity, while pasteurization tends to produce a more consistent decline in this parameter. A study on broccoli juice reported that thermal treatment decreased DPPH values, whereas thermosonication increased antioxidant activity compared to pasteurisation. This supports the present results (59). This result is also consistent with findings reported for parsley juice (60).

In Figure 7C, the TFC was determined as 34.47 ± 0.94 mg CE/L for CON, 36.64 ± 0.23 mg CE/L for TS, and 32.53 ± 0.58 mg CE/L for PAS. No significant differences were observed between CON–TS and CON–PAS (*p* > 0.05; ns), whereas the TS–PAS comparison revealed significantly higher values for TS (*p* < 0.05). Similarly, in Figure 7D, the total anthocyanin content (TAC) was highest in the TS group (24.25 ± 0.59 mg C_3_GE/100 mL). The CON group (22.5 ± 0.49 mg C3GE/100 mL) remained close to TS, with no significant difference between the groups (*p* > 0.05; ns). However, PAS showed a reduced TAC value (19.8 ± 0.36 mg C_3_GE/100 mL); the TS–PAS comparison was significant at p < 0.01, while the CON–PAS comparison was significant at *p* < 0.05. Overall, the combined evidence from Figures 7A–D indicates that thermosonication can be considered a processing method that preserves and, to some extent, enhances bioactive compounds and antioxidant capacity. In contrast, pasteurization appears to be associated with greater losses, particularly in heat-sensitive compounds such as phenolics and anthocyanins. In a study on blackberry juice subjected to different temperature conditions during thermosonication, it was reported that higher temperatures negatively affected TAC values (61). Exposure to higher thermosonication temperatures may reduce the levels of certain bioactive compounds due to thermal degradation. The level of applied acoustic energy can induce fluctuations in antioxidant capacity, resulting in either increases or decreases depending on processing intensity. The physical effects generated during sonication disrupt cell structure, promoting the release of bioactive constituents from the food matrix and potentially enhancing their bioaccessibility. However, under high-temperature, high-power conditions, intensified cavitation can occur, and the collapse of cavitation bubbles may generate reactive species, for example, hydroxyl radicals. These reactive species can contribute to the oxidative degradation of phenolic compounds (62). In a study by Yıkmış et al. (63) on pomegranate juice, thermosonication was reported to preserve bioactive compounds, consistent with the findings of the present study.

### 3.7 *In silico* molecular docking analysis

Table 8 shows the molecular docking insights of α-amylase in complex with the selected compounds. The reactive components of vinegar, such as phenolic compounds, may determine the overall effect through molecular-level interactions. According to the α-amylase inhibition study of CON, TS, and PAS production methods, TS has a statistically significantly higher inhibitory activity on the enzyme over the others. To identify potential molecular players in inhibition, phenolic compound analysis was performed in our study, and phenolic compounds with distinct quantities were examined in molecular docking studies to elucidate probable intermolecular interactions. Molecular docking is a computational approach for predicting the binding modes and affinities of ligands within protein targets, with a primary focus on non-covalent interactions. The molecular docking studies provided significant insights into the inhibitory mechanisms of the selected phenolic compounds against α-amylase (PDB ID: 1HNY). The active site, comprising the catalytic triad amino acids D197, E233, and D300, is vital for the enzyme’s catalytic activity; therefore, an inhibitory ligand likely binds near the catalytic site, inducing a thermodynamic or conformational change that blocks other ligands’ binding to the enzyme.

**Table 8:** Molecular docking insights of alpha amylase in complex with the chosen compounds.

1HNY docked with the compound below	Docking score of the best pose	Favorably interacting aminoacids
Quercetin	−8.654	Trp59, Tyr62, Gln63, Arg195, Glu233, Asp300
Gallic acid	−5.81	Tyr62, Glu233, Asp300
Trans-cinnamic acid	−7.988	Gln63, Glu233, Ile235, His299, Asp300

**Figure 8:**
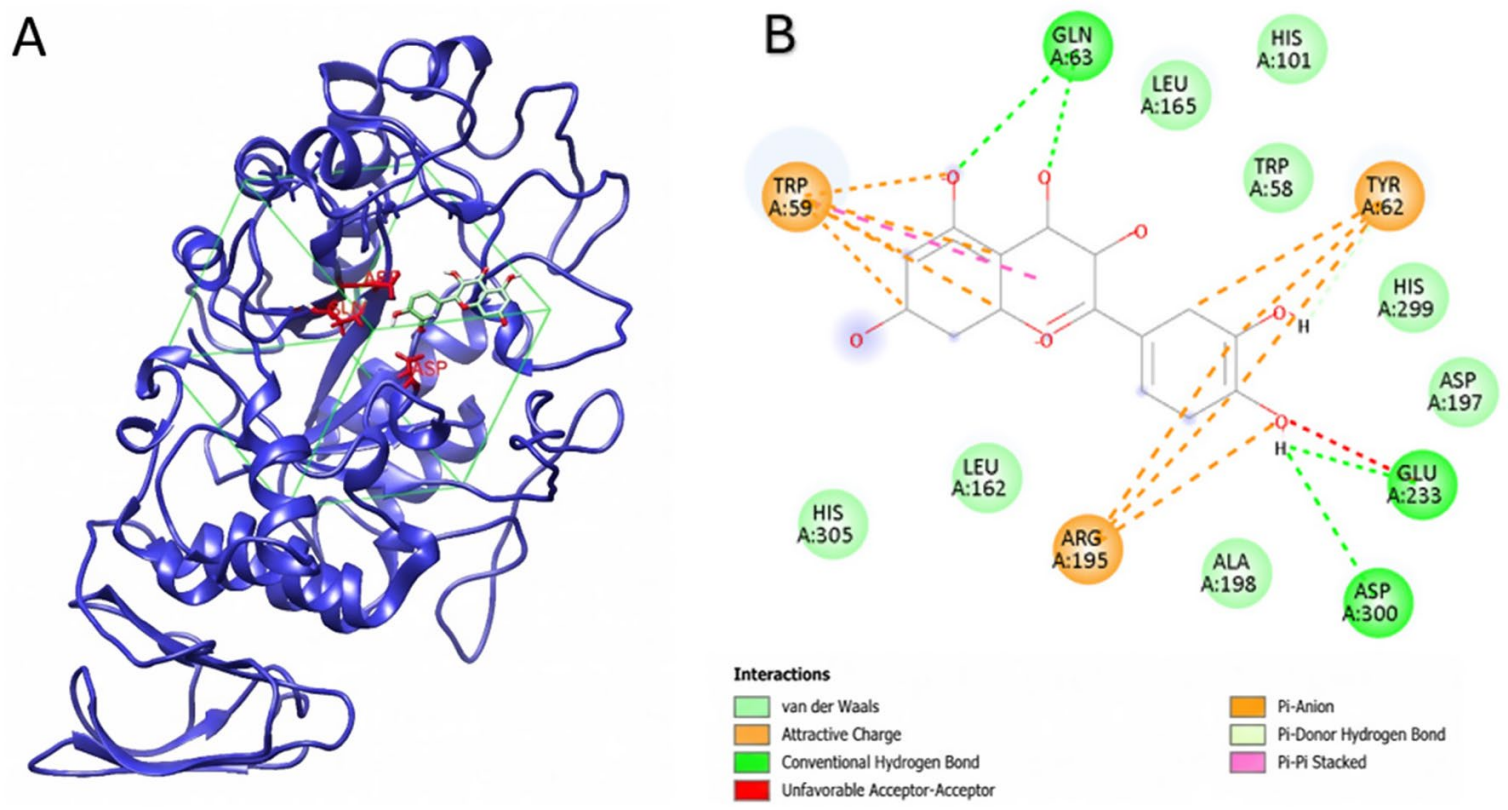
Molecular docking outcomes of phenolic ligands with pancreatic α-amylase (PDB: 1HNY). The active site comprising the catalytic triad amino acids D197, E233, and D300 is shown in red. Gridbox is shown in green. **(A)** Highest-ranking docking pose of quercetin bound to 1HNY (docking score: −8.654). **(B)** Interaction map of the α-amylase–quercetin complex highlighting hydrogen bonds (green dashed arrows) and *π*–π interactions (green dashed lines).

Based on phenolic compound analysis, quercetin was found in poppy vinegar produced via the TS method; by contrast, vinegar produced via neither the CON nor the PAS method contains quercetin. Thus, quercetin may contribute to α-amylase inhibition. To understand quercetin’s affinity for α-amylase, molecular docking studies were conducted as a supporting analysis. With a docking score of −8.654 kcal/mol, the strong interaction between quercetin and α-amylase is primarily attributed to a robust network of favorable interactions with key residues, including Trp59, Tyr62, Gln63, Arg195, and most importantly, the catalytic residues Glu233 and Asp300 (Figure 8). Due to the presence of quercetin in the poppy vinegar produced via TS, the observed higher inhibition rates may be attributed to steric hindrance at the α-amylase catalytic site and the favorable interactions between the ligand and key catalytic residues. According to the docking poses and interaction diagrams, the catalytic amino acids comprising the catalytic site (D197, E233, D300) showed van der Waals and hydrogen-bond interactions. This strengthens the possibility of quercetin interacting with the catalytic site of α-amylase and resulting in enzymatic inactivation.

**Figure 9:**
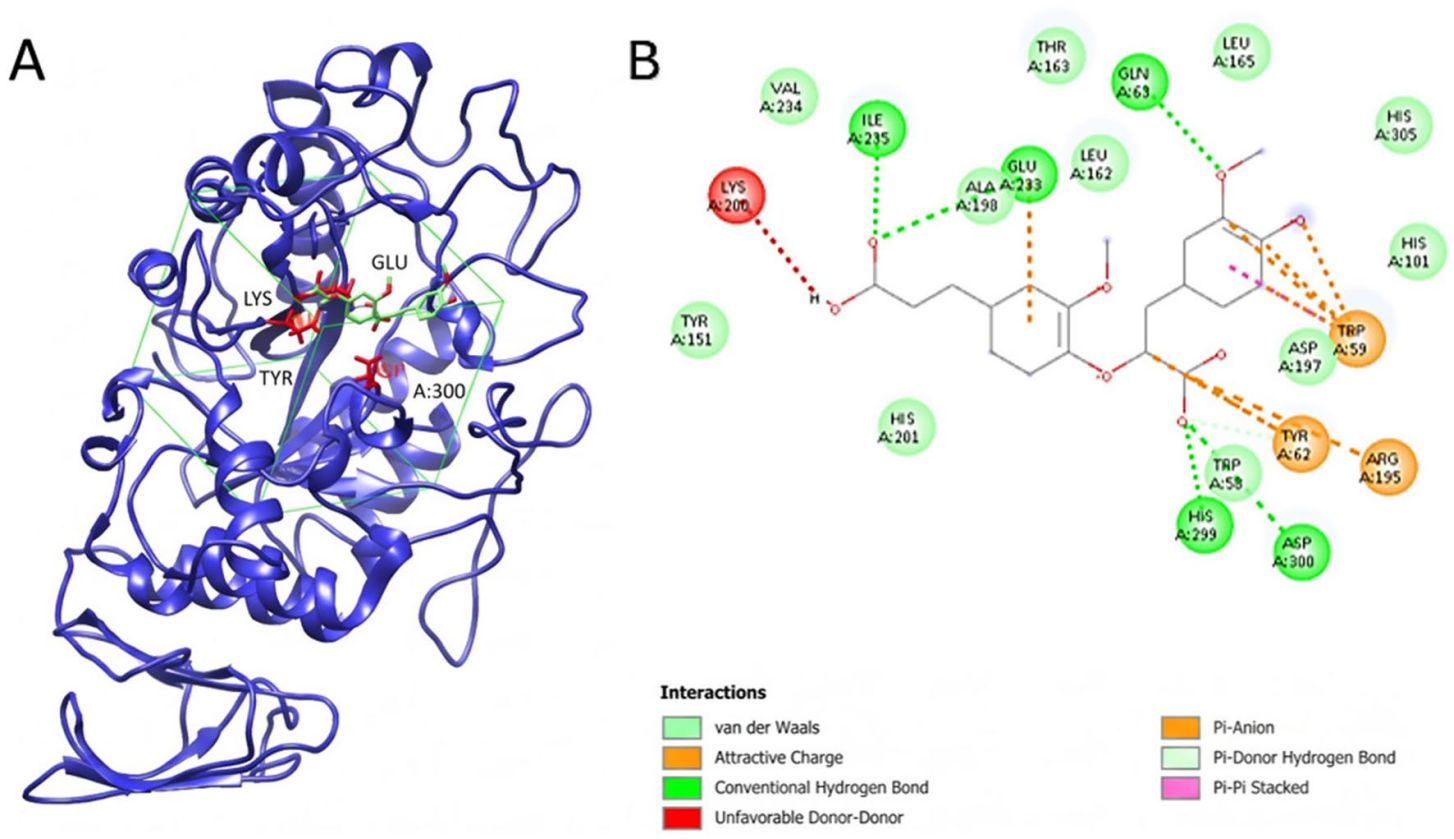
Molecular docking outcomes of phenolic ligands with pancreatic α-amylase (PDB: 1HNY). The active site comprising the catalytic triad amino acids D197, E233, and D300 is shown in red. Gridbox is shown in green. **(A)** Highest-ranking docking pose of *trans*-cinnamic acid bound to 1HNY (docking score: −7.988). **(B)** Interaction map of the α-amylase–*trans*-cinnamic acid complex showing hydrogen bonds (green dashed arrows), π–π interactions (pink dashed lines), and an unfavorable interaction between Lys200 and *trans*-cinnamic acid (red dashed line).

Contrarily, based on the phenolic compound analysis, trans-cinnamic acid was not detected in TS-produced poppy vinegar, while it was detected in CON and PAS-produced vinegar as 0.18 and 0.95 μg/mL, respectively. CON and PAS did not differ significantly in α-amylase inhibition; however, CON inhibition was slightly greater. It might be thought that the trans-cinnamic acid amount is inversely related to the inhibition potential of the vinegar type. The α-amylase and trans-cinnamic acid docking study showed a high affinity score of −7.988 kcal/mol; however, it also revealed an unfavorable donor–donor interaction between Lys200 and the hydrogen atom of trans-cinnamic acid, probably due to the positive character of both molecules (Figure 9).

**Figure 10:**
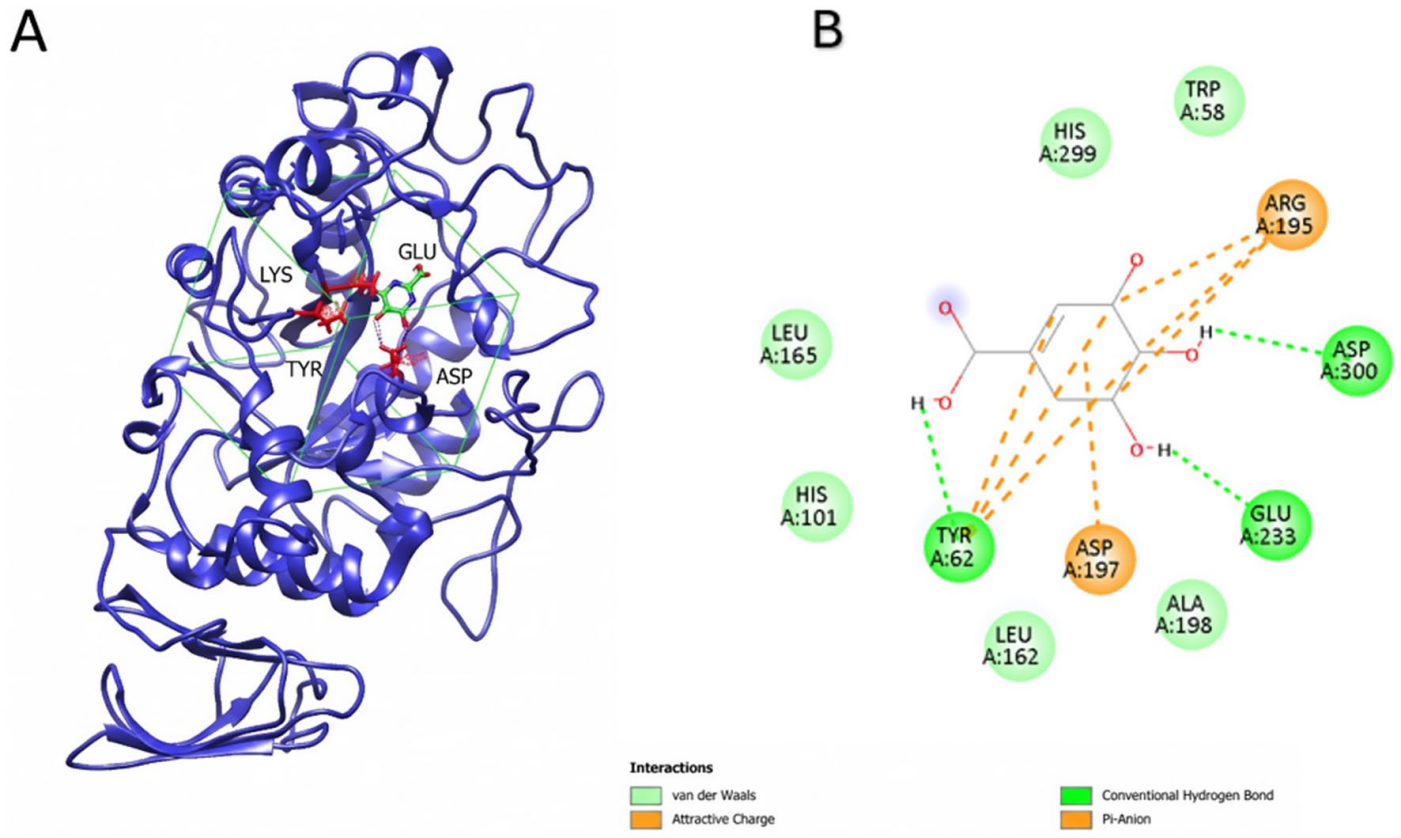
Molecular docking outcomes of phenolic ligands with pancreatic α-amylase (PDB: 1HNY). The active site comprising the catalytic triad amino acids D197, E233, and D300 is shown in red. Gridbox is shown in green. **(A)** Highest-ranking docking pose of gallic acid bound to 1HNY (docking score: **−**5.842). **(B)** Interaction map of the α-amylase–gallic acid complex, highlighting hydrogen bonds (green dashed arrows).

Gallic acid quantity based on the phenolic compound analysis indicated a decrease for PAS and a slight increase (statistically non-significant) for TS with respect to CON methods. Gallic acid, being a smaller phenolic molecule, yielded a lower binding score of −5.81 kcal/mol, yet it successfully targeted the critical residues Glu233 and Asp300, which are essential for the enzyme’s hydrolytic activity (Figure 10). These findings suggest that the stability of the protein-ligand complexes may be largely governed by interactions with the catalytic center, with quercetin offering the most favorable inhibitory profile, while the molecular docking analysis is consistent with the α-amylase enzymatic activity test.

Acarbose is a standard compound employed for diabetes treatment and used as a cognate ligand for re-docking and method validation purposes (46). According to the re-docking studies, the acarbose docking score was −9.636, which is comparable to our study’s docking results. Based on the in silico study results, quercetin shows a strong interaction with the 1HNY active site, while gallic acid shows a moderate-to-high affinity for the enzyme. Thus, even further verification is needed by in silico (e.g., simulation) and *in vitro* experiments; it could be hypothesized that quercetin and gallic acid affinity towards the α-amylase catalytic site may play a role in enzyme inhibition.

## 4 Conclusion

The results of this study demonstrated that thermosonication provides a suitable treatment range for poppy vinegar to preserve bioactive fractions and support functional efficacy, as indicated by the evaluated chemical parameters and in vitro bioactivity outputs. The combination of multivariate experimental design and machine-learning-based modeling enabled reliable characterization of relationships between treatment parameters and enzyme inhibition responses, while multi-objective optimization identified conditions that maximized the targeted biological activity. The relatively better preservation of total phenolic content, flavonoid levels, and radical-scavenging capacity in thermosonicated samples may be attributed to cavitation-assisted mass transfer at moderate temperatures and to the enhanced release of soluble bioactives. Although changes in individual phenolic compounds suggest that the treatment influences the biomolecular composition, in the absence of compound–activity correlations, regression modeling, or analyses of synergistic interactions, these findings should be interpreted as associative rather than causal. Similarly, molecular docking analysis supported the mechanistic plausibility of enzyme–ligand interactions but did not establish direct quantitative causality for the observed *in vitro* inhibition responses; moreover, as docking was performed only for α-amylase due to structural and methodological constraints, the molecular basis of α-glucosidase inhibition remains to be further elucidated. From a practical application perspective, the findings offer significant value for both the food industry and functional food development, as the optimized thermosonication conditions (13.86 min, 84.68% amplitude, 47.40 °C) provide a reliable operational window for industrial-scale production, while also demonstrating improved preservation of heat-sensitive phenolics and enhanced antidiabetic potential compared to conventional pasteurization. Consequently, thermosonication represents a promising green processing alternative for producing high-quality, bioactive-rich traditional vinegars that may serve as functional dietary components targeting postprandial glycemic control. However, because microbiological inactivation and storage stability were not evaluated, definitive conclusions regarding microbial safety and shelf-life extension cannot be drawn. Therefore, future studies should focus on industrial validation, in vitro digestion, bioavailability/bioaccessibility, shelf-life assessment, and comprehensive enzyme-level mechanistic analyses.

## Data Availability

The raw data supporting the conclusions of this article will be made available by the authors, without undue reservation.
