# Supplementary material for: Thermosonication of traditional poppy vinegar: modulation of biomolecules, phenolic profile, and antidiabetic potential in a functional fermented food
Source: Front Nutr. 2026 Apr 30;13:1828372. doi: 10.3389/fnut.2026.1828372 (PMC13171545; doi:10.3389/fnut.2026.1828372)
Supplement: Supplementary file 1 [file Supplementary_File_1.docx]

**Supplementary Information**

**HPLC method validation (brief)**

Method validation was conducted to support comparative quantification of phenolic compounds. LOD and LOQ were defined at signal-to-noise (S/N) ratios of 3 and 10, respectively. Accuracy was evaluated by spike-recovery at three fortification levels (low/mid/high; n = 3) and expressed as % recovery. Repeatability (intra-day precision) was assessed using replicate injections/analyses (n = 3) and reported as %RSD for peak areas and/or concentrations.

**Representative chromatograms**


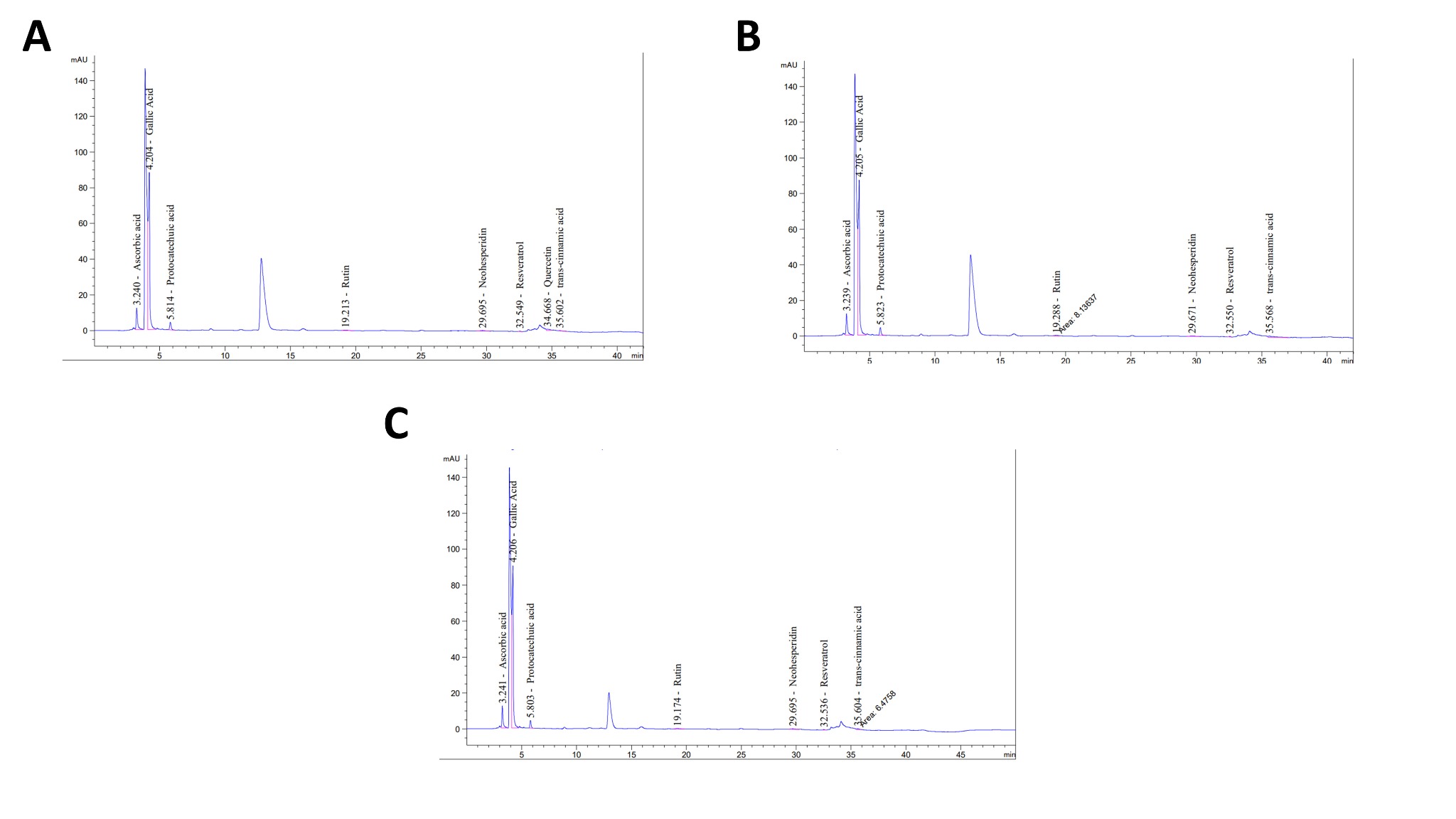


**Figure S1.** Representative HPLC–DAD chromatograms of poppy vinegar samples: (A) Control (CON), (B) Pasteurized (PAS), and (C) Thermosonicated (TS)

**Molecular Docking Method Validation**

1HNY structure is used in the molecular docking studies since the protein in its apo form and the crystal has a great resolution; moreover this structure is abundantly used in the literature. However, this structure does not contain a cognate ligand for re-docking and method validation. In order to validate our methods; we used another human α-amylase structure (PDB ID:1B2Y) which contains acarbose as a cognate ligand. Firstly, 1HNY and 1B2Y structures aligned to show the similarity (RMSD= 0.131 Å) and to have the same space coordinates for the prefixed gridbox. According to the alignment, 1HNY and 1B2Y exactly matches and could be used interchangable by fixing the space coordinates. The cognate ligand acarbose is a branched carbohydrate and occupy a larger broad with respect to quercetin, gallic acid and transcinnamic acid that are analyzed in our study; thus, same coordinates with a larger box size (X:10, Y:50, Z:18 is remained with the box size 30:30:30 instead of 20:20:20 for re-docking). Acarbose was re-docked to 1B2Y with a docking score -9.636. The RMSD of less than 2 Å (0.650 Å) of the acarbose validated our protocol.


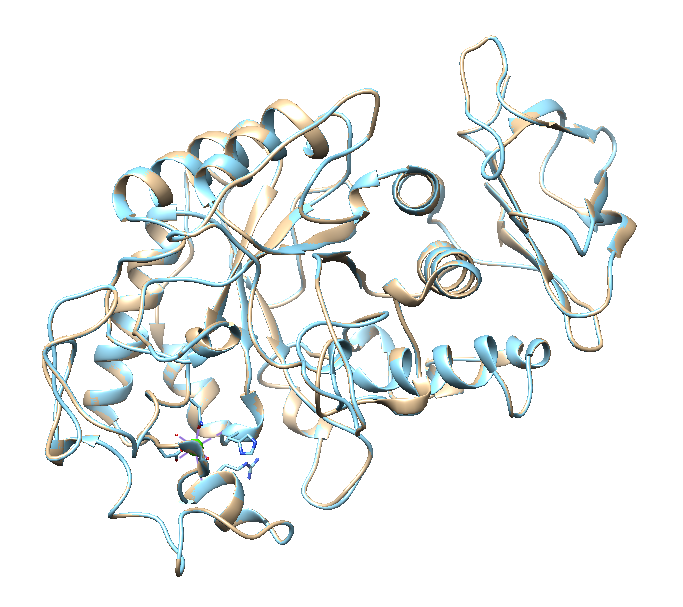


Figure S2. 1HNY crystal and 1B2Y crystal aligned with an RMSD of 0.131 Å


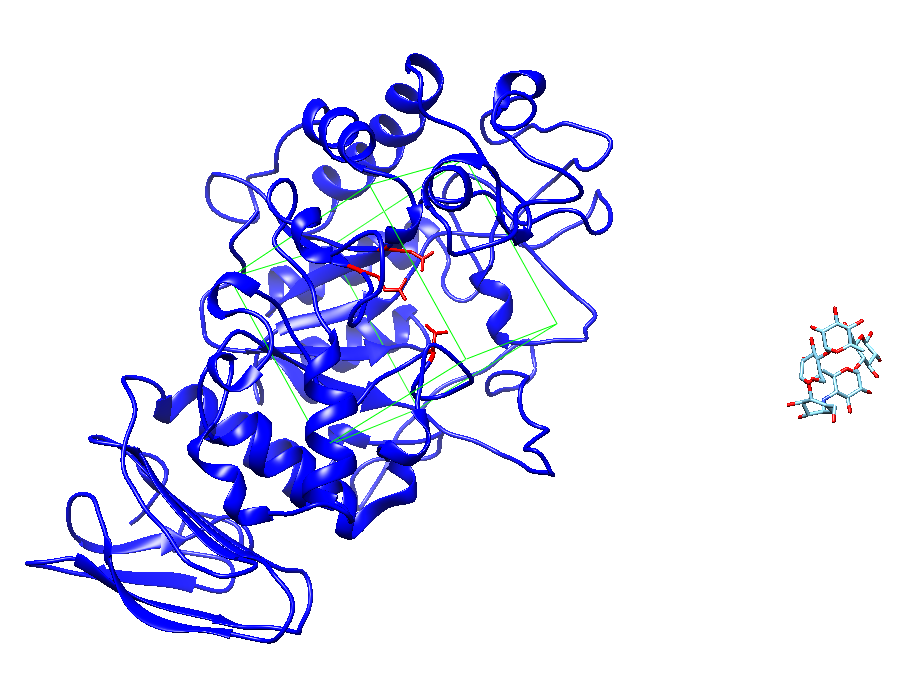


Figure S3. α- Amylase structure (1B2Y) with the gridbox including the active site aminoacids (D197, E233 and D300) and the cognate ligand acarbose prepared for docking for method validation.


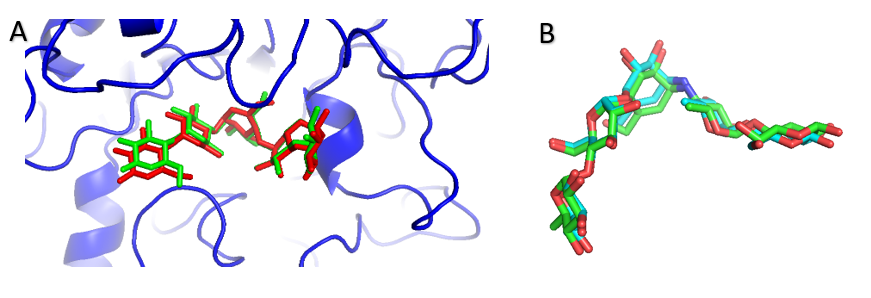


Figure S4. α- Amylase re-docked with the cognate ligand acarbose with a docking score -9.636 and RMSD is calculated as 0.650 by using PyMOL for docking method validation.
